# Long Noncoding RNA LINC00941 Links Oncogenic KRAS Signaling to Aggressiveness, DNA Repair, and Chemoresistance in Pancreatic Cancer

**DOI:** 10.1002/iub.70140

**Published:** 2026-11-09

**Authors:** Thalita Bueno Corrêa, Gabriel Lucas da Fonseca, Rauni Borges Marques, Sandro Mascena Gomes‐Filho, Lutero Augusto Hasenkamp, Yuli Thamires Magalhães, Lillian Cristina Russo Vieira, Nicolas Paulino Rezende, Stefany Cristine Rodrigues da Silva, Nicolas Carlos Hoch, Fabio Luis Forti, Elena Levantini, Eduardo Moraes Reis, Daniela Sanchez Bassères

**Affiliations:** ^1^ Biochemistry Department Chemistry Institute, São Paulo University São Paulo Brazil; ^2^ Interunit Graduate Program in Bioinformatics, University of São Paulo São Paulo Brazil; ^3^ Institute of Biomedical Technologies, National Research Council (CNR) Pisa Italy

**Keywords:** chemoresistance, DNA repair, KRAS, long noncoding RNA, pancreatic cancer

## Abstract

Oncogenic KRAS mutations are the primary drivers of pancreatic ductal adenocarcinoma (PDAC), but the noncoding landscape downstream of KRAS remains to be fully elucidated. Here, by integrating PDAC RNA sequencing and exome data from The Cancer Genome Atlas (TCGA), we identified 49 long intergenic noncoding RNAs (lincRNAs) differentially expressed according to KRAS mutation status. Among these, LINC00941 emerged as a primary candidate, as its expression is regulated by KRAS in both loss‐ and gain‐of‐function models and is significantly upregulated in PDAC tumors. Analysis of single‐cell RNA sequencing and clinical datasets (TCGA/ICGC) revealed that LINC00941 is significantly enriched in PDAC malignant epithelial cells and correlates with poor PDAC patient prognosis. Functional assays demonstrated that LINC00941 knockdown impairs PDAC cell migration and invasion. Notably, LINC00941‐depleted cells exhibit delayed DNA repair following irradiation, as shown by the slower resolution of radiation‐induced DNA strand breaks, and increased sensitivity to gemcitabine‐induced apoptosis. Transcriptomic and RT‐qPCR analyses associated the loss of LINC00941 with a significant reduction in key DNA repair and survival‐related genes, including BACH1, TOP2A, and BIRC5. These findings suggest that LINC00941 is required to maintain genomic stability and promote survival under therapeutic stress in KRAS‐driven cells. In conclusion, our study identifies a novel KRAS/LINC00941 axis that supports a pro‐survival and chemoresistant phenotype in PDAC, positioning LINC00941 as a mediator of the DNA damage response and a potential therapeutic target in PDAC.

## 1 Introduction

Pancreatic ductal adenocarcinoma (PDAC) is the most common malignancy of the pancreas, accounting for approximately 90% of cases and is an extremely lethal disease, with a median survival of less than 1 year and a 5‐year overall survival rate of less than 15% [1, 2, 3]. This dismal prognosis stems from an inability to detect PDAC in early stages, its aggressive metastatic dissemination, and a lack of effective targeted therapies. The most common molecular mechanism that drives PDAC growth and maintenance is the constitutive activation of the KRAS GTPase by oncogenic mutations, which are widespread in PDAC, occurring in over 90% of patients [4]. Even though direct KRAS inhibitors have been recently approved for KRAS^G12C^‐mutant lung cancer [5], KRAS^G12C^ mutations are found in less than 2% of PDAC cases [6], and to date, there are no specific *KRAS*‐driven therapies approved for PDAC patients [4]. In addition, PDAC tumors often display intrinsic or acquired resistance to conventional chemotherapy, as well as to KRAS inhibitors [4, 6, 7]. To overcome these challenges, it is crucial to decipher the molecular mechanisms employed by KRAS to promote malignant transformation, metastatic spread, and chemoresistance. Identifying new downstream effectors could reveal potential new KRAS targets that could be explored in combination with KRAS inhibitors, not only for the development of new antitumor therapeutic approaches for PDAC, but also to serve as biomarkers for early PDAC detection and disease monitoring.

Beyond its role as the initiating oncogenic event, KRAS mutational status and mutant allele dosage profoundly shape the global transcriptional landscape of PDAC. Although the small subset of KRAS wild‐type PDACs frequently harbors alternative alterations that activate the RAS–MAPK pathway, such as BRAF mutations or kinase gene fusions [8, 9], these tumors display gene expression programs distinct from those of KRAS‐mutant tumors, including reduced expression of classical subtype markers [9]. This suggests that the effects of oncogenic KRAS on gene expression extend beyond the activation of MAPK signaling per se. Among KRAS‐mutant tumors, increased mutant KRAS dosage drives early tumor progression, metastasis, and the emergence of aggressive, undifferentiated phenotypes [10], is associated with the basal‐like transcriptional subtype [11], and predicts poor overall survival [12]. Moreover, acute suppression of mutant KRAS in PDAC cells triggers extensive, largely ERK‐dependent transcriptional reprogramming [13]. However, these studies have focused predominantly on protein‐coding genes, and how KRAS mutational status affects the noncoding transcriptome of PDAC remains poorly understood.

Long noncoding RNAs (lncRNAs) comprise a broad class of RNA molecules longer than 200 nucleotides that do not code for proteins. LncRNAs can fold into diverse, complex secondary and tertiary structures capable of interacting with DNA, RNAs, and proteins. As a result, lncRNAs play a wide array of important biological roles [14, 15]. In the context of cancer, the expression of lncRNAs is often dysregulated, and aberrantly expressed lncRNAs have been linked to all major cancer hallmarks and clinical outcomes, with a subset displaying bona fide oncogenic or tumor suppressive functions [16, 17, 18]. Specifically, lncRNAs have been implicated in the maintenance of genomic stability and the DNA damage response (DDR), biological hallmarks that are frequently hijacked by cancer cells to promote survival under therapeutic stress [19]. Beyond their functional roles, lncRNAs exhibit high tissue specificity and are typically expressed at lower levels than mRNAs, making them promising candidates for precision therapeutic intervention with reduced off‐target toxicity [15]. Furthermore, their stability in biological fluids highlights their potential as noninvasive biomarkers for disease monitoring [15, 20]. However, while oncogenic *KRAS* is the primary driver of PDAC, the specific lncRNA effectors that mediate its downstream effects on tumor progression and therapy resistance remain largely unidentified.

In this study, we integrated bulk and single‐cell transcriptomics with clinical datasets to characterize the noncoding landscape downstream of KRAS in PDAC. We identified LINC00941 as a novel KRAS‐regulated lncRNA that is significantly enriched in malignant epithelial cells and serves as a predictor of poor patient outcome. Through loss‐ and gain‐of‐function experiments, we demonstrate that LINC00941 is a downstream effector of oncogenic KRAS signaling that promotes genomic stability and confers gemcitabine resistance. Our findings establish a previously undescribed KRAS/LINC00941 axis that supports PDAC aggressiveness, positioning this lncRNA as a potential therapeutic target to overcome chemoresistance in pancreatic cancer.

## 2 Materials and Methods

### 2.1 Differential Expression Analysis According to KRAS Mutation Status in PDAC Gene Expression Datasets

PDAC RNA‐seq data from TCGA (PAAD‐US, 145 cases) and ICGC (PACA‐AU, 98 cases) were retrieved from the ICGC data portal (https://dcc.icgc.org), and corresponding *KRAS* mutation data for both datasets were retrieved from cBioPortal (RRID:SCR_014555), based on whole‐exome sequencing mutation calls. Differential expression analysis was performed as previously described [21]. Briefly, TCGA raw read counts were aligned to the GRCh38.p13 genome using RSEM [22] and normalized in R. DESeq2 [23] was used to compare *KRAS* mutant (KRAS^mut^, *n* = 131) versus wild‐type (KRAS^WT^, *n* = 14) samples. Differentially expressed genes were filtered for intergenic lncRNAs (lincRNAs) with log_2_ fold change ≥ |1| and adjusted *p* value ≤ 0.05.

For downstream analyses, CPM‐normalized counts were transformed using log_2_(CPM + 1) and used to compare lincRNA expression between KRAS^mut^ and KRAS^WT^ samples in both datasets. Statistical significance was determined using the nonparametric Wilcoxon rank‐sum test, with *p* < 0.05 considered significant.

### 2.2 Differential lncRNA Expression Analysis in PDAC Tumors and Adjacent Pancreatic Tissue (APT)

We have previously performed a comprehensive lncRNA differential expression analysis on a dataset comprised of 14 PDAC patient‐matched tumor and nontumor samples [24]. Therefore, for this analysis, we retrieved the differential expression data for the lincRNAs identified in this study. A comprehensive description of the differential expression analysis performed for this dataset can be found in Paixão et al. [24].

### 2.3 PDAC Single‐Cell lincRNA Expression Analysis

A processed count matrix with single‐cell RNA‐seq expression data from 41,986 cells from 24 PDAC and 15,544 cells from 11 nontumor pancreatic samples generated by Peng et al. [25] was retrieved from the Genome Sequence Archive (accession number CRA001160). The original data were already filtered, and only cells with more than 200 detected genes and < 10% mitochondrial RNA content were kept. The “Seurat” package (v.5.0.3) in R [26] was used for normalization, selection of informative features, scaling, dimensionality reduction, and clustering of cells into different cellular identities. Clusters were annotated using the cell markers described by Peng et al. [25]. The normalized log‐transformed count frequency in individual cells in each cluster was used to compare expression of LINC00941 and co‐expressed genes among the different cell clusters. Enrichment dot plots were generated with the Seurat “DotPlot” function. In these plots, each gene is represented by two dots (Ductal 1 and Ductal 2). Dot size corresponds to the percentage of cells in the cluster with nonzero expression of the gene, and dot color represents the scaled average expression (enrichment score), ranging from −0.4 (low) to +0.4 (high). Differential expression analysis between Ductal 1 and Ductal 2 epithelial cells was performed using the Wilcoxon method. Genes were considered differentially expressed if they showed a log_2_ fold change ≥ |0.58| and an adjusted *p* value ≤ 0.05.

### 2.4 LINC00941 Co‐Expression and Functional Enrichment Analysis

RNA‐seq count data from the TCGA‐PAAD‐US cohort were analyzed to identify genes co‐expressed with LINC00941. Raw gene‐level counts were imported into R and processed with edgeR (v4.4.2). Genes with missing values were excluded, and counts per million (CPM) were calculated. Lowly expressed genes were filtered out using filterByExpr function in edgeR. LINC00941 passed the filtering threshold and was retained. Filtered counts were variance‐stabilized by log_2_ transformation [log_2_(CPM + 1)]. The expression profile of LINC00941 was extracted, and pairwise Spearman correlation coefficients were computed between LINC00941 and all remaining genes. Correlation testing was performed with the *cor.test* function in R, and *p* values were adjusted for multiple comparisons using the Benjamini–Hochberg false discovery rate (FDR) method. Genes with FDR‐adjusted *p* values < 0.05 were considered significantly correlated with LINC00941. The top 300 genes most strongly correlated with LINC00941 (based on Spearman correlation coefficients) were selected for functional enrichment analysis. Gene set enrichment was performed using the Enrichr R package (v3.4), considering annotations from gene ontology [27], KEGG [28], and Reactome [29] databases. A significance threshold of an adjusted *p* value of < 0.05 was used to select enriched terms.

### 2.5 Survival Analysis

PDAC cases with incomplete clinical information regarding vital status, overall survival, or follow‐up time were excluded from survival analysis, after which 87 cases from the ICGC‐PACA‐AU dataset and 141 cases from the TCGA‐PAAD‐US dataset remained for analysis. For each sample, raw read counts from Ensembl‐annotated genes (version 89) were normalized to CPM units. Survival analysis was performed as previously described [21]. Briefly, Kaplan–Meier (KM) survival curves were generated using the R survival package [30]. For each gene of interest, patients were grouped into two expression groups (high and low), according to the median expression across all samples. Differences in KM survival curves of high and low expression groups were inferred by the log‐rank test with a significance threshold of *p* < 0.05. Hazard ratios (HRs) with 95% confidence intervals were estimated using Cox regression and refer to the relative risk of death in the high expression group.

### 2.6 Quantitative Real‐Time PCR (qRT‐PCR)

Total RNA was isolated using TRIzol reagent (Invitrogen) following the manufacturer's protocol. RNA concentration and purity were assessed by spectrophotometry (*A*_260_/*A*_280_ ratio) using a NanoDrop spectrophotometer (Thermo Fisher Scientific), and genomic DNA was removed by DNase I treatment (Qiagen) prior to reverse transcription. RNA (1 or 0.5 μg) was used to synthesize cDNA in a reaction volume of 20 μL using the Superscript III first‐strand synthesis system (Thermo Fisher Scientific) with provided oligo(dT)/random hexamer primers, and diluted fivefold to a final volume of 100 μL. Real‐time PCR analysis using 3 μL of the cDNA was performed in a GeneAmp 5700 Sequence Detection System (Applied Biosystems) using 6 μL of a SYBR Green master mix (Thermo Fisher Scientific) and 400 nM of gene‐specific forward and reverse primers designed using Primer3web version 4.0.0 tool (http://bioinfo.ut.ee/primer3/). Primer sequences, RefSeq transcript identifiers, and amplicon sizes are listed in Supplementary Table 1. Reactions were run in a final volume of 12 μL, in technical triplicate, under the following cycling conditions: 95°C for 10 min, followed by 40 cycles of 95°C for 15 s and 60°C for 1 min. Amplification specificity was confirmed by melting‐curve analysis, and no‐template controls and no‐reverse‐transcription controls were included in each run. Amplification efficiencies were determined from standard curves of serially diluted cDNA and ranged from 90% to 110%. Relative quantitation was determined by the ΔΔCt method using GAPDH or HMBS as endogenous controls. Samples in which no amplification was detected within 40 cycles were assigned a Ct value of 40 (one APT sample for LINC00941; Figure 3A); because tumor and APT samples were compared with the rank‐based Wilcoxon test, the choice of this imputed value did not affect the statistical result.

### 2.7 Cell Lines and Cell Culture

Cell passages were kept to a minimum, and none of the cells were passaged continuously for more than 6 months. Human pancreatic cancer cell lines harboring *KRAS* mutations, AsPC‐1 (*KRAS* ^G12D^; ATCC CRL‐1682; RRID:CVCL_0152) and PANC1 (*KRAS* ^G12D^; ATCC CRL‐1469; RRID:CVCL_0480), were grown in DMEM (Invitrogen Corp) supplemented with 10% (vol/vol) fetal bovine serum (FBS). For gain‐of‐function experiments, we used the immortalized, nontumorigenic human pancreatic ductal epithelial cell line HPDE (HPDE6‐E6E7 Clone 6, also known as H6c7), which was established by transduction of normal human pancreatic duct epithelial cells with the E6 and E7 genes of human papillomavirus 16 [31], and its isogenic derivative HPDE‐KR, which stably expresses oncogenic *KRAS* ^G12V^ [32]. HPDE and HPDE‐KR cells were kindly provided by Dr. Albert S. Baldwin (Lineberger Comprehensive Cancer Center, University of North Carolina at Chapel Hill, NC, USA). Because HPDE and HPDE‐KR cells share the same genetic background and differ in the expression of oncogenic KRAS, this isogenic pair allows the effect of oncogenic KRAS on gene expression to be assessed independently of the additional genetic alterations present in PDAC cell lines. HPDE and HPDE‐KR isogenic cells were grown in Keratinocyte‐SFM supplemented with EGF and BPE (ThermoFisher).

### 2.8 Patient Samples (Internal Validation Set)

The internal validation set consisted of seven primary PDAC samples and five nonmalignant APT samples obtained from 12 different patients (i.e., tumor and APT samples were not paired) who underwent surgical resection at the A.C. Camargo Cancer Center (São Paulo, Brazil). Clinical data were available for 9 of the 12 patients (5 men and 4 women; median age: 75 years; range: 65–81 years). KRAS mutational status of the tumor samples was determined by targeted next‐generation sequencing of genomic DNA (20 ng input; Illumina) using a multi‐gene amplicon panel (Ampli14v3) covering hotspot regions including KRAS Codons 12, 13, 61, 117, and 146 and BRAF Codon 600. Coverage depth at KRAS codon 12 was > 1.8 × 10^6^ reads. KRAS mutations (G12D, G12R, or G12V; variant allele frequencies of 1%–15%) were detected in four of the five sequenced tumor samples, and one tumor sample was KRAS wild type and harbored an in‐frame BRAF deletion (Supplementary Table 6); two tumor samples were not sequenced. The clinical characteristics of the internal validation set are summarized in Supplementary Table 6. Samples were snap‐frozen in liquid nitrogen immediately after resection and were provided by the A.C. Camargo Cancer Center Biobank. The study was approved by the Research Ethics Committee of the A.C. Camargo Cancer Center (opinion number 457.476; CAAE 15059213.0.0000.5432), written informed consent was obtained from all participants prior to sample collection, and the study complied with the Declaration of Helsinki.

### 2.9 siRNA Transfections

The cells were seeded in six‐well plates at a density of 1 × 10^5^ cells/well 24 h before transfection. siRNA transfections were performed with 4 μL of the Lipofectamine 3000 transfection reagent and 50 nM of either a nontargeting siRNA control or siRNA SMART pools targeting LINC00941 or KRAS (siGENOME siRNA, Dharmacon), according to the manufacturer's instructions. The cells were collected for extraction of RNA and protein at 48, 72, or 96 h after transfection. For migration and invasion analyses, the cells were collected 96 h after transfection.

### 2.10 Western Blotting

Whole‐cell lysates of siRNA‐transfected cells were prepared 72 h post‐transfection using RIPA buffer (20 mM Tris–HCl pH 7.5, 150 mM NaCl, 1 mM Na_2_EDTA, 1 mM EGTA, and 1% NP‐40) containing protease and phosphatase inhibitors (1 μg/mL leupeptin, 1% sodium deoxycholate, 2.5 mM sodium pyrophosphate, 1 mM β‐glycerophosphate, and 1 mM Na_3_VO_4_). Aliquots of total lysate (30 μg protein/lane) were separated in 12% or 15% polyacrylamide minigels using a MiniPROTEAN vertical electrophoresis cell (Bio‐Rad). Electrophoresis was conducted with running buffer containing 25 mM Tris, 190 mM glycine, and 0.1% SDS at 120 V for 60–90 min. Next, proteins were transferred to nitrocellulose membranes (Bio‐Rad) using Towbin buffer (25 mM Tris, 192 mM glycine, and 20% methanol) at 350 mA for 2 h and 30 min. The blots were blocked with 5% BSA in TBST (20 mM Tris, pH 7.5, 150 mM NaCl, and 0.1% Tween‐20) for 1 h and incubated with primary antibodies, followed by incubation with horseradish peroxidase (HRP)‐conjugated secondary antibodies. Chemiluminescence detection was performed using a ChemiDoc MP Imaging System (Bio‐Rad). The following primary antibodies were used: anti‐GAPDH (Santa Cruz Biotechnology), anti‐β‐tubulin (Santa Cruz Biotechnology), and anti‐PanRAS (Merck Millipore). All primary antibodies were used at a dilution of 1:1000 in TBST, containing 5% BSA and 0.1% NaN_3_, and incubated for 16 h at 4°C. The following secondary antibodies were used at a dilution of 1:3500 in TBST and incubated for 1 h at room temperature: anti‐rabbit IgG HRP (Promega) or anti‐mouse IgG HRP (Promega).

### 2.11 Transwell Migration and Invasion Assays

Transwell assays were performed using uncoated (migration assay) or Matrigel‐coated (invasion assay) 24‐well transwell inserts with 8 μm pore size membrane filters (Corning). AsPC‐1 cells were transfected with siRNAs as described, and 72 h post‐transfection, 1 × 10^5^ AsPC‐1 cells were resuspended in 300 μL of serum‐free medium and added to the upper chamber of the transwell insert. Complete medium (500 μL) was added to the lower chamber as a chemoattractant, and the cells were incubated for 24 h at 37°C in 5% CO_2_. Nonmigrating cells were removed from the upper surface of the membrane using a cotton swab, after which migrating/invading cells on the bottom surface were stained with crystal violet. Images were obtained under an IX51 Inverted Microscope (Olympus). The cells from three random fields from three independent experiments were counted using ImageJ software.

### 2.12 Cell Death Assay

Cell death was examined using a propidium iodide (PI, Sigma‐Aldrich) and Hoechst 33342 (HO, Sigma‐Aldrich) double‐staining method. AsPC‐1 cells were transfected with siRNA as described and, 24 h post‐transfection, were seeded on 96‐well plates at a density of 1 × 10^4^ cells/well. For siRNA‐only experiments, the cells were analyzed after 48 h to assess the effect of LINC00941 knockdown on viability in the absence of gemcitabine. For chemoresistance experiments, gemcitabine (10, 20, or 100 μM) was added 24 h after plating, and cell death was quantified after 5 days. For PI and HO staining, the cells were washed three times with PBS, incubated with HO (25 μg/mL) and PI (1 μg/mL) for 10 min at 37°C, and examined by fluorescence microscopy. Images were acquired with a custom TissueFAXS i‐Fluo (TissueGnostics) system on a Zeiss AxioObserver 7 microscope using 20× Plan‐Neofluar (NA 0.5) or 40× Plan‐Apochromat (NA 0.95) objectives and an ORCA Flash 4.0 v3 (Hamamatsu) camera. The percentage of dead cells was determined as the fraction of PI‐positive cells among all HO‐stained cells. For each condition, ≥ 6 × 6 adjacent fields were acquired, and ≥ 3000 cells were analyzed in triplicate using StrataQuest (TissueGnostics). For the siRNA‐only experiment (no gemcitabine), percent cell death in siCTRL versus si941 samples was compared using a paired *t*‐test. For chemoresistance experiments, percent cell death was quantified for siCTRL and si941 samples treated with gemcitabine at 0, 10, 20, or 100 μM. To address replicate‐to‐replicate variability and enable different biological interpretations, data were normalized in two complementary ways: (i) to the siCTRL group at each gemcitabine dose and replicate, which isolates the siRNA effect independent of gemcitabine‐induced cytotoxicity, and (ii) to the 0 μM gemcitabine condition within each siRNA group, which provides a baseline for assessing relative sensitization. Statistical testing was performed with linear mixed‐effects models including siRNA and gemcitabine concentration as fixed effects and replicate as a random effect. ANOVA on the mixed model tested for main and interaction effects; residuals were examined for normality (Shapiro–Wilk) and homogeneity of variance (Levene's test). When assumptions were violated, a nonparametric aligned rank transform (ART) ANOVA was applied. Estimated marginal means (EMMs) were derived from the mixed model, and Tukey‐adjusted pairwise contrasts between si941 and siCTRL were performed at each gemcitabine dose. Fold‐change values of si941 relative to siCTRL were calculated from the EMMs. In all analyses, differences were considered statistically significant at *p* < 0.05.

### 2.13 DNA Damage and Repair Assay

siRNA‐treated AsPC‐1 cells (6 × 10^4^) were seeded in 12‐well plates in duplicate, incubated for 72 h, and subjected to ionizing gamma irradiation at a dose of 10 Gy. Irradiated cells were kept in culture for recovery for 15 min, 30 min, 1 h, 2 h, or 4 h and subsequently processed for alkaline comet assays. Nonirradiated control cells were processed in parallel. For the comet assays, the cells were trypsinized, resuspended in 0.5% (w/v) low‐melting agarose, layered on top of glass slides previously prepared with a thin layer of 1.5% (w/v) agarose, covered with coverslips to ensure homogeneous spread of cells over the slide surface, and incubated overnight at 4°C in lysis solution (2.5 M NaCl, 100 mM EDTA, and 10 mM Tris pH 10) for membrane disruption. Next, samples were subjected to DNA denaturation (30 min, 4°C) and subsequent electrophoresis in alkaline pH buffer (300 mM NaOH, 1 mM EDTA, pH > 13) at 25 V and 300 mA for 30 min at 4°C. After electrophoresis, the slides were immersed in neutralization solution (0.4 M Tris, pH 7.5) for 15 min, followed by fixation in absolute ethanol for 5 min and staining with EtBr (20 μg/mL) for DNA visualization. Slide images were captured in a fluorescence microscope Olympus BX51 and analyzed using Andor Komet 6.0 software (Oxford Instruments Inc.), which measures the amount of genomic DNA damage expressed as “Olive tail moment units,” that is, the product of the tail length and the fraction of total DNA in the tail. A total of 100 cells were examined in each condition/time point (50 cells/replicate).

### 2.14 Statistical Analysis

All values are presented as mean ± SD or as representative images from independent experiments, with the number of replicates indicated in figure legends. For pairwise comparisons, Student's *t*‐test was used for normally distributed data and the Wilcoxon rank‐sum test for non‐normally distributed data. In gene expression analyses, comparisons of fold‐change (2^−ΔΔC*t*^) values were analyzed accordingly, with effect sizes reported as Cohen's *d* (for *t*‐tests) or rank‐biserial correlation (for Wilcoxon tests). For comparisons involving more than two groups, differences were assessed by one‐way ANOVA, followed by Bonferroni's multiple comparison test, unless otherwise specified. Statistical significance was defined as *p* < 0.05. Cell lines, antibodies, software, and databases used in this study, together with their research resource identifiers (RRIDs), are listed in Supplementary Table 7.

## 3 Results

### 3.1 Identification of Differentially Expressed Long Intergenic Noncoding RNAs (lincRNAs) According to *KRAS* Mutation Status

To identify differentially expressed lincRNAs in PDAC based on KRAS mutation status, we analyzed publicly available RNA sequencing (RNA‐seq) and exome sequencing data from TCGA (*n* = 145). Our analysis compared PDAC patients harboring KRAS mutations (*n* = 131) with those exhibiting wild‐type KRAS (*n* = 14). Among the 131 KRAS‐mutant tumors, the most frequent alterations were G12D (*n* = 58; 44.3%), G12V (*n* = 38; 29.0%), and G12R (*n* = 25; 19.1%), followed by Q61H (*n* = 6; 4.6%), Q61R (*n* = 2; 1.5%), G12C (*n* = 1; 0.8%), and G13C (*n* = 1; 0.8%), consistent with the distribution of KRAS alleles previously reported in PDAC [8] (Supplementary Table 5). To refine our analysis, we focused specifically on lincRNAs, noncoding transcripts that do not overlap annotated protein‐coding regions, eliminating potential ambiguities arising from overlapping coding sequences. Differential expression was determined using a threshold of log_2_ fold change ≥ |1| and an adjusted *p* value ≤ 0.05. This approach identified 49 differentially expressed lincRNAs, including 21 upregulated and 28 downregulated lincRNAs in KRAS‐mutant patients (Figure 1A and Supplementary Table 2). For validation, we selected the 10 most abundantly expressed lincRNAs based on their mean normalized counts across all samples (Supplementary Table 2). Among these, nine were significantly upregulated in KRAS‐mutant patients (LINC01133, LINC01559, RP3‐340N1.2, RP11‐211G23.2, LINC00941, RP11‐362F19.1/LINC02432, AC006262.5, RP5‐884M6.1, and RP11‐470L19.2), while MIR3142HG was downregulated in the DESeq2 analysis, although this difference was not significant by the Wilcoxon test on normalized counts (Figure 1A,B).

**FIGURE 1:**
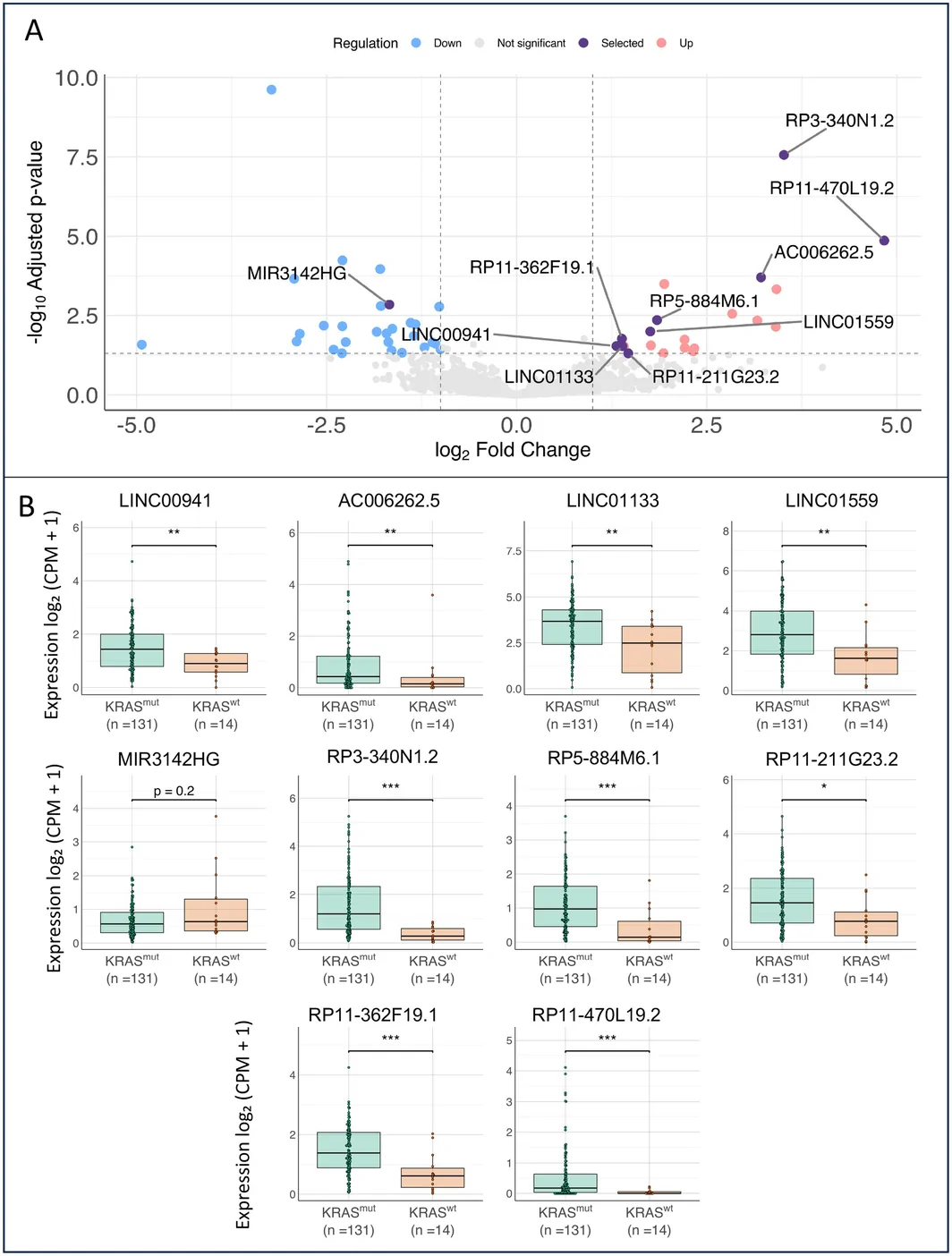
Identification of differentially expressed lincRNAs according to *KRAS* mutation status. (A) Volcano plot of lincRNA expression changes in the TCGA PDAC cohort. The *x*‐axis shows the log_2_ fold change (log_2_FC, KRAS^MUT^ vs. KRAS^WT^), and the *y*‐axis shows the –log_10_ adjusted *p* value. Dotted lines indicate the thresholds for significance (log_2_FC ≥ |1|, adjusted *p* < 0.05). Genes are colored as significantly upregulated (up), significantly downregulated (down), not significant, or highlighted as selected abundant lincRNAs. (B) Dot plots comparing log_2_‐transformed CPM‐normalized counts of the indicated lincRNAs in KRAS^MUT^ (*n* = 131) and KRAS^WT^ (*n* = 14) tumors in the TCGA PDAC cohort (PAAD‐US, *n* = 145). Statistical significance was assessed using two‐tailed Wilcoxon rank‐sum tests, with significance levels denoted as *p < 0.05, ** *p* < 0.01 and *** *p* < 0.005.

To validate these findings in an independent dataset, we examined the expression of these candidates in the ICGC PDAC cohort (PACA‐AU, *n* = 98), stratifying patients by KRAS mutation status (Supplementary Figure 1). In this cohort, which includes a smaller number of KRAS wild‐type samples (*n* = 9) and was sequenced from total RNA libraries [33], several candidates changed in the same direction as in the TCGA cohort, but none of the differences reached statistical significance. Among the upregulated candidates, LINC00941 (two‐tailed Wilcoxon *p* = 0.089) and AC006262.5 (*p* = 0.138) showed the lowest *p* values for higher expression in KRAS‐mutant tumors. Based on the consistent direction of change in both cohorts, LINC00941 and AC006262.5 were selected for further analyses.

We started by investigating whether LINC00941 and AC006262.5 correlated with the malignant transformed phenotype of PDAC tumors. To do so, we analyzed RNA‐seq data previously generated in our laboratory, which included paired tumor and adjacent nontumor tissue samples from 14 PDAC patients, previously described by da Paixão et al. [24]. Both LINC00941 and AC006262.5 exhibited significantly higher expression in tumor samples compared to adjacent nontumor tissue (Figure 2A).

**FIGURE 2:**
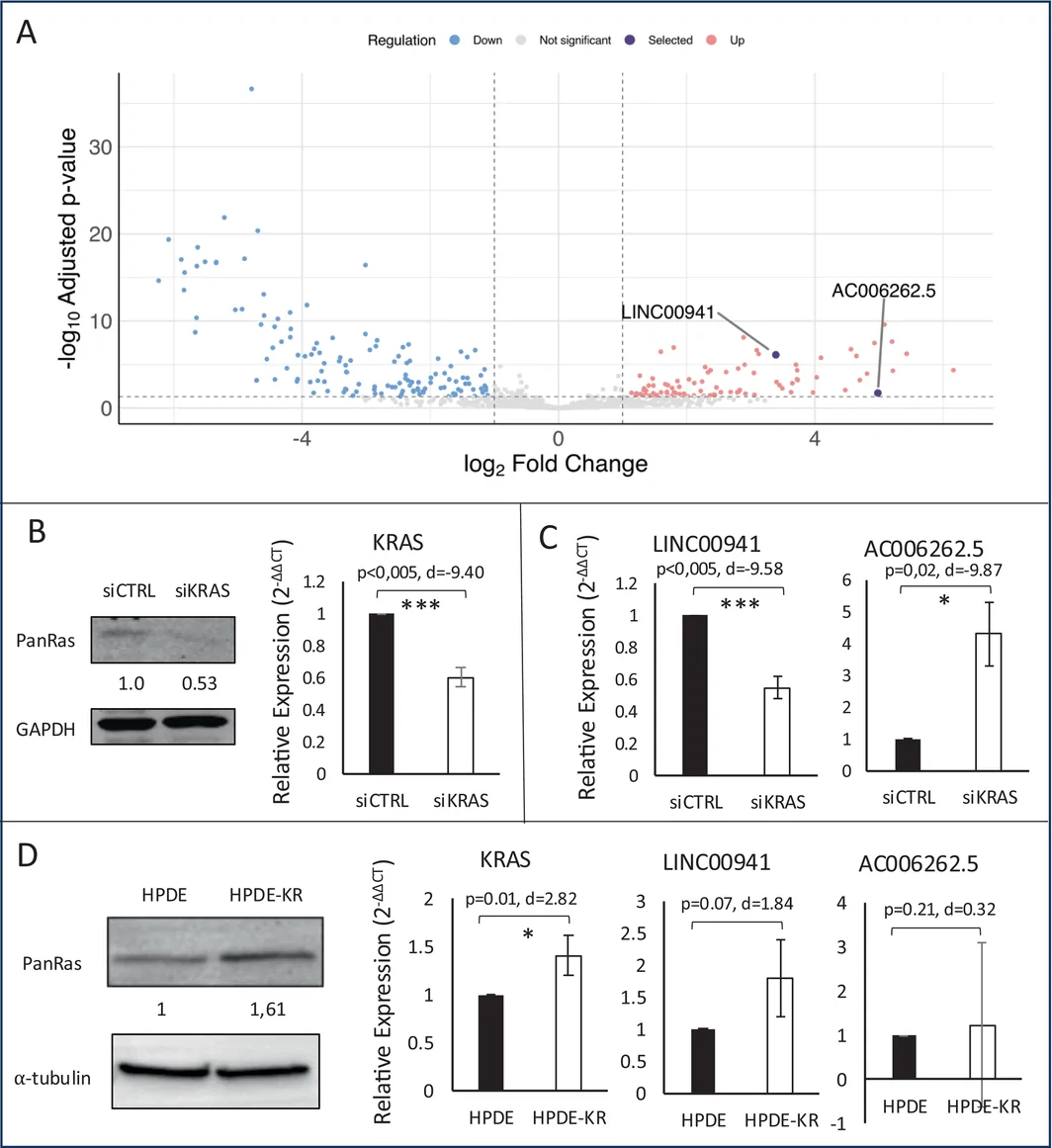
KRAS regulates the expression of lincRNAs in PDAC cell lines. (A) Volcano plot of lincRNA expression changes in paired PDAC tumors and adjacent pancreatic tissue (APT, *n* = 14). The *x*‐axis represents log_2_ fold change (log_2_FC, PDAC vs. APT), and the *y*‐axis represents –log_10_ adjusted *p* value. Dotted lines indicate the thresholds for significance (log_2_FC ≥ |0.58|, adjusted *p* < 0.05). Genes are colored by significance status (upregulated, downregulated, not significant), with LINC00941 and AC006262.5 highlighted. (B) Validation of KRAS knockdown in AsPC‐1 cells by RNA interference. *Left*: Western blot comparing Ras protein levels in siCtrl and siKRAS groups (representative of *n* = 3). GAPDH served as a loading control. *Right*: qRT‐PCR analysis of KRAS mRNA expression in siKRAS and siCtrl groups (*n* = 3). (C) KRAS‐dependent regulation of candidate lincRNAs in AsPC‐1 cells. qRT‐PCR analysis of lincRNA expression in siKRAS and siCtrl groups (*n* = 3). (D) Expression of KRAS and lincRNAs in isogenic pancreatic cells. *Left*: Western blot comparing Ras protein levels in HPDE and HPDE‐KR cells (representative of *n* = 1). α‐Tubulin served as a loading control. *Right*: qRT‐PCR analysis of KRAS mRNA expression in HPDE and HPDE‐KR cells (*n* = 3). HMBS was used as an endogenous control for all qRT‐PCR experiments. Statistical significance for qRT‐PCR data was assessed using a paired one‐tailed Student's *t*‐test for KRAS knockdown validation and a paired two‐tailed Student's *t*‐test for target lincRNA regulation. Significance levels are denoted as *p < 0.05, ***p < 0.005. Exact *p* values and Cohen's *d* effect sizes are indicated.

### 3.2 LINC00941 Is a KRAS Target in AsPC‐1 Cells

To investigate whether KRAS regulates LINC00941 and AC006262.5 expression in PDAC, we used siRNA‐mediated knockdown of KRAS in AsPC‐1 cells. Compared to control cells transfected with a nontargeting siRNA (siCtrl), the cells transfected with a KRAS‐specific siRNA SMARTpool (siKRAS) exhibited a ~50% reduction in total RAS protein levels (Figure 2B). This was corroborated by qPCR, which revealed a ~50% decrease in KRAS mRNA levels (Figure 2B), confirming effective knockdown. As expected, KRAS knockdown significantly reduced LINC00941 expression by 46% (Figure 2C). However, contrary to our hypothesis, AC006262.5 expression increased significantly following KRAS suppression (Figure 2C).

To assess whether these observations were influenced by the genetic background of AsPC‐1 cells, we analyzed expression of the lincRNA candidates in isogenic low‐passage immortalized human pancreatic ductal epithelial (HPDE) cells and their KRAS‐transformed counterparts (HPDE‐KR). Despite a 41% increase in KRAS expression in HPDE‐KR cells, AC006262.5 expression in HPDE‐KR cells was highly variable and did not differ significantly from HPDE controls. In HPDE‐KR cells, LINC00941 expression was on average ~80% higher than in HPDE controls (Cohen's *d* = 1.84). Although this difference did not reach the conventional significance threshold (*p* = 0.07), the effect size was large and, given the limited statistical power of experiments with three biological replicates, we interpret this result as supportive evidence, as it agrees in direction with the significant decrease in LINC00941 expression observed upon KRAS knockdown in AsPC‐1 cells (Figure 2D).

Collectively, LINC00941 expression decreased upon KRAS knockdown and increased upon oncogenic KRAS expression, consistent with its regulation by KRAS signaling.

### 3.3 LINC00941 Is Overexpressed in PDAC Tumors and in Malignant PDAC Cells and Is Associated With Worse PDAC Prognosis

To further investigate the association between LINC00941 and the malignant phenotype, we analyzed its expression in seven PDAC patient samples and five nontumor pancreatic tissue samples from our internal validation set (see Section 2 and Supplementary Table 6) by qPCR. LINC00941 expression was higher in PDAC than in nontumor samples (Figure 3A). Although this difference did not reach the conventional significance threshold (Wilcoxon rank‐sum test, *p* = 0.073, effect size *r* = 0.54), the effect size was large and, given the small number of samples, we interpret this result as supportive evidence, as it agrees in direction with the significant upregulation of LINC00941 in PDAC observed in our RNA‐seq data (Figure 2A). One tumor sample (56T) was classified as pancreatic intraepithelial neoplasia in the analyzed fragment (Supplementary Table 6); excluding this sample did not change the direction of the difference, and the effect size remained large (*p* = 0.13; *r* = 0.50).

**FIGURE 3:**
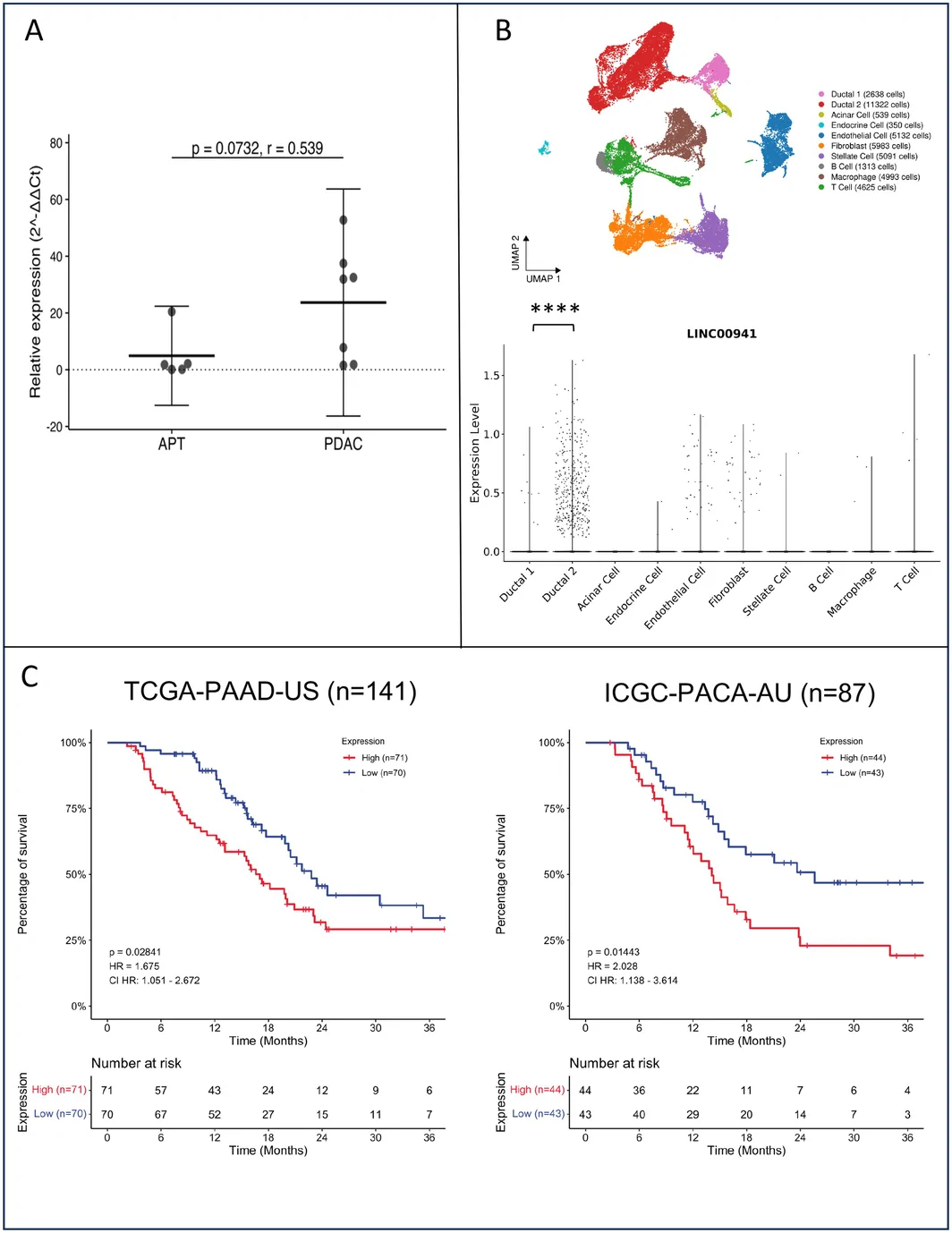
LINC00941 is overexpressed in PDAC tissue and is associated with PDAC patient survival. (A) qRT‐PCR analysis of LINC00941 expression in PDAC tumors (*n* = 7) compared with adjacent pancreatic tissue (APT, *n* = 5). Results are shown as dot plots of relative expression values, with horizontal bars indicating group means. HMBS was used as an endogenous control. Statistical significance was assessed using the Wilcoxon rank‐sum test; exact *p* values and rank‐biserial effect sizes are indicated. (B) Single‐cell RNA‐seq analysis of LINC00941 expression in PDAC. *Upper*: Uniform manifold approximation and projection (UMAP) plot of annotated cell clusters identified with Seurat. The number of cells per cluster is indicated. *Lower*: Violin plot showing normalized log‐transformed expression level of LINC00941 in individual cells (dots) across clusters. Because most cells show no detectable LINC00941 expression, the violins appear collapsed at zero. Differential expression testing was performed on normalized, log_2_‐transformed counts using the Wilcoxon rank‐sum test with *p* values adjusted for multiple testing (Benjamini–Hochberg), and significance level denoted as ****padj < 0.001. (C) Kaplan–Meier survival analysis based on LINC00941 expression in the TCGA (PAAD‐US, *n* = 141) and ICGC (PACA‐AU, *n* = 87) cohorts. Patients were stratified into high‐ and low‐expression groups using the median expression cutoff. Differences in survival were evaluated using the log‐rank test. Log‐rank *p* values and hazard ratios (HRs) with 95% confidence intervals are shown.

Although LINC00941 was identified in bulk tumor samples, which contain not only cancer cells but also other cell types, such as stromal and immune cells, it is essential to confirm its enrichment in the malignant cell population. This is particularly relevant for PDAC, given its highly infiltrative nature, where tumors often include a substantial proportion of stromal components and normal pancreatic tissue [34].

To determine whether LINC00941 is specifically expressed in malignant PDAC cells, we analyzed single‐cell RNA‐seq data (GSA, accession number CRA001160) from 24 PDAC tumor samples and 11 control pancreatic tissue samples, pre‐clustered into 10 distinct cell types [25]. While LINC00941 expression was detected in endothelial cells and fibroblasts, it was significantly enriched in Type 2 ductal cells in contrast to Type 1 ductal cells (Figure 3B). Notably, whereas Type 1 ductal cells were found both in control pancreatic samples and in PDAC tumor samples and found to correspond to normal ductal cells, Type 2 ductal cells were exclusively present in tumor samples and identified by Peng et al. [25] as the malignant PDAC cell population. These findings indicate that LINC00941 is preferentially expressed in malignant PDAC cells.

More importantly, high LINC00941 expression correlates with reduced overall survival in PDAC patients from both the TCGA and ICGC cohorts (Figure 3C). These findings reinforce LINC00941 as the most promising KRAS‐associated lincRNA candidate likely to be involved in promoting malignant tumor behavior.

### 3.4 LINC00941 Depletion Reduces Migration and Invasion of KRAS‐Mutant PDAC Cells

To investigate whether LINC00941 contributes to the KRAS‐driven malignant phenotype, we silenced LINC00941 in AsPC‐1 cells using siRNA and assessed its impact on cell viability, migration, and invasion (Figure 4). LINC00941 knockdown was validated, revealing a 63% reduction in LINC00941 expression (Figure 4A). LINC00941 knockdown did not increase cell death (Figure 4B). Nonetheless, LINC00941 depletion decreased cell migration and invasion by 21% and 38%, respectively (Figure 4C,D). These findings suggest that LINC00941 enhances tumor progression by promoting a more aggressive phenotype.

**FIGURE 4:**
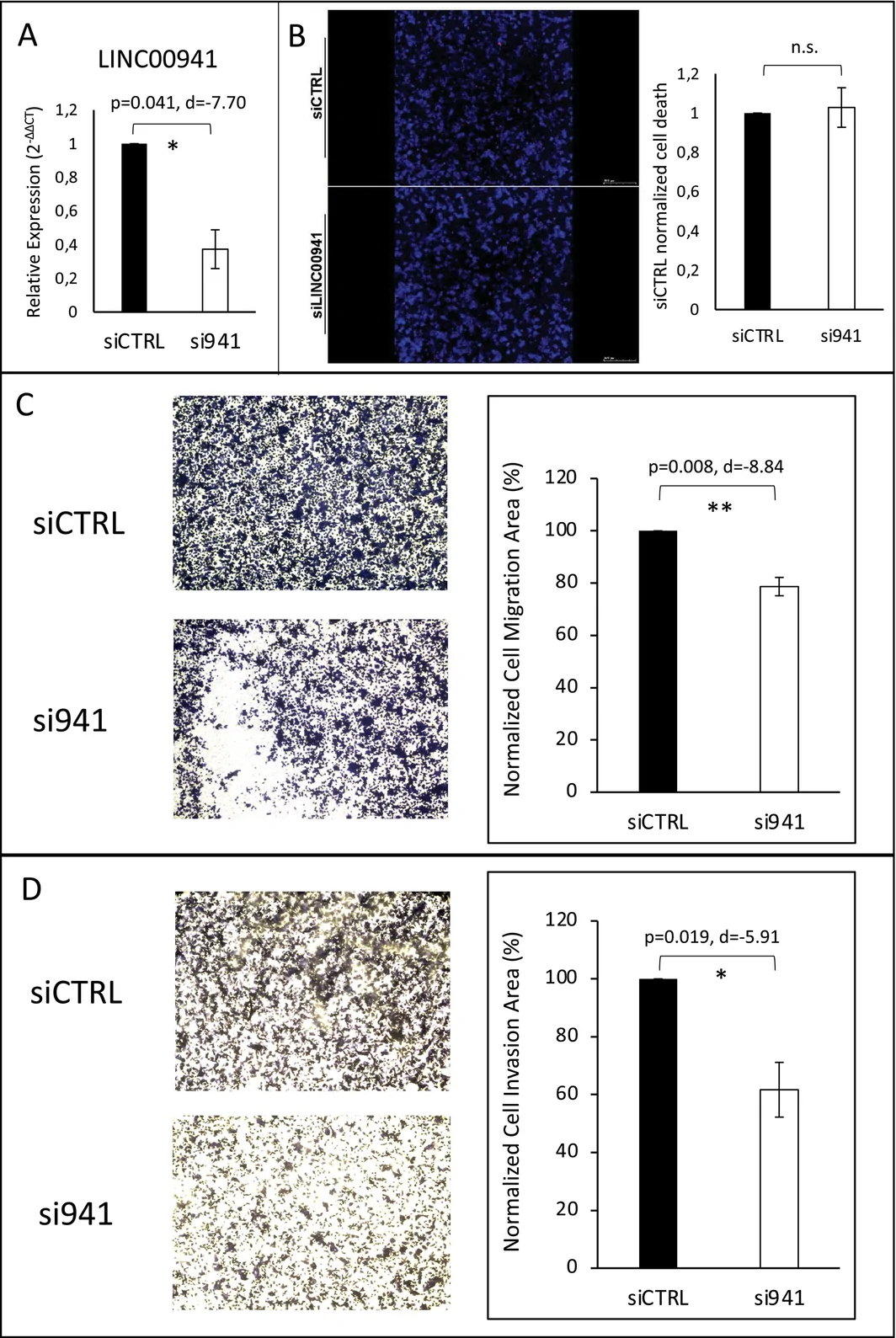
Targeting LINC00941 reduces migration and invasion of KRAS‐mutant AsPC‐1 cells. AsPC‐1 cells were transfected with a nontargeting control siRNA (siCTRL) or a SMARTpool siRNA targeting LINC00941 (si941). (A) qRT‐PCR analysis of LINC00941 expression in siCTRL and si941 groups (*n* = 2). Data represent fold‐change relative to siCTRL. HMBS was used as an endogenous control. (B) Cell viability analysis. *Left*: Representative images of Hoechst‐ and PI‐stained cells (*n* = 3). *Right*: Quantification of normalized cell death relative to siCTRL (*n* = 3). (C, D) Transwell migration (C) and invasion (D) assays. *Left*: Representative images (*n* = 3). *Right*: Quantification shown as mean ± SD. Statistical significance was assessed by paired Student's *t*‐tests (one‐tailed for panel A; two‐tailed for panels B–D). Significance thresholds: *p* < 0.05 (*), *p* < 0.01 (**), n.s., not significant. For significant comparisons, exact *p* values and Cohen's *d* effect sizes are reported. Comparisons are indicated by horizontal bars.

### 3.5 LINC00941 Depletion Delays the Resolution of Radiation‐Induced DNA Strand Breaks

LncRNAs are frequently functionally related to protein‐coding genes with which they are co‐expressed [35, 36]. Using Weighted Gene Co‐expression Network Analysis (WGCNA) of RNA‐seq data from PDAC tumors and adjacent normal tissues, we previously identified LINC00941 to be co‐expressed with protein‐coding genes associated with DNA repair [24]. This module comprises genes from several DNA repair pathways, including homologous recombination (HR), non‐homologous end joining, nucleotide excision repair, and single‐strand break (SSB)/base excision repair (e.g., POLB, PARP2, and MBD4).

To validate this association, we examined the expression of DNA repair‐related genes with high connectivity to the DNA repair module of co‐expressed transcripts that included LINC00941 [24], including genes whose expression changes in PDAC in the same direction as LINC00941 (higher in tumors: SPIDR, PPP4C, BACH1, and ATRX) and genes that change in the opposite direction (lower in tumors: TERF2IP and CDK7). We also included TOP2A, a DNA repair gene found to be positively correlated with LINC00941 in a different WGCNA module of the same data. LINC00941 knockdown significantly downregulated PPP4C, BACH1, and TOP2A. ATRX (*p* = 0.07, Cohen's *d* = −2.98) and SPIDR (*p* = 0.18, *r* = −1.00) were not significantly changed, while TERF2IP (*p* = 0.53) and CDK7 (*p* = 0.42) expression remained unchanged (Figure 5A). Thus, LINC00941 knockdown reduced the expression of genes that change in the same direction as LINC00941 in PDAC, but not those that change in the opposite direction, thus suggesting that LINC00941 does not seem to act to repress gene expression, but rather acts as a positive regulator of gene expression. The LINC00941‐mediated regulation of PPP4C and BACH1 was confirmed in PANC‐1 cells (Supplementary Figure 2). Interestingly, analysis of single‐cell sequencing data of PDAC tumors revealed that, of the DNA repair genes positively correlated with LINC00941, only the qPCR‐validated genes (BACH1, PPP4C, and TOP2A) are enriched in malignant Type 2 ductal cells, with ATRX and SPIDR being enriched in Type 1 ductal cells (Figure 5B).

**FIGURE 5:**
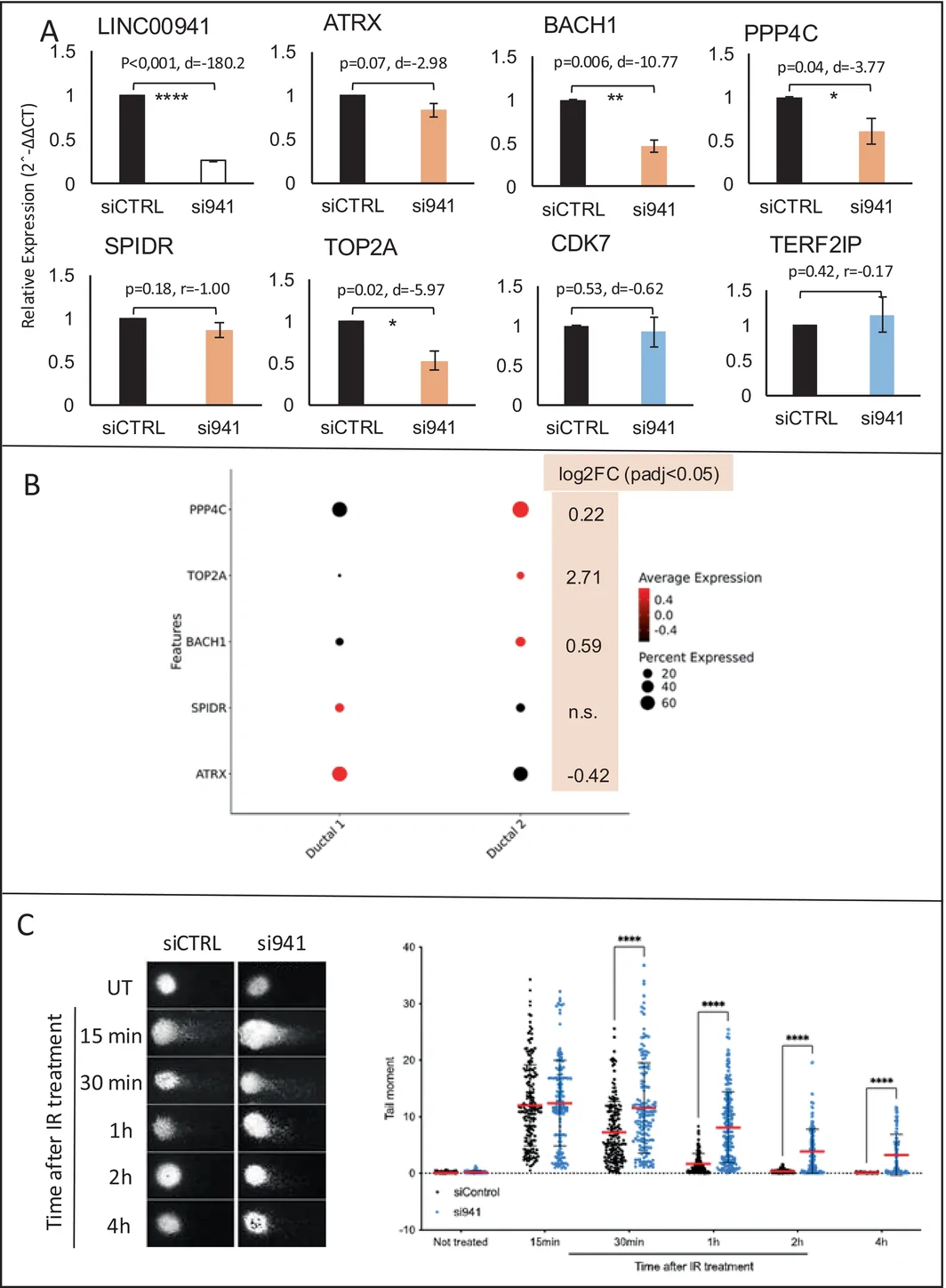
LINC00941 depletion delays the resolution of radiation‐induced DNA strand breaks. AsPC‐1 cells were transfected with nontargeting control siRNA (siCTRL) or a SMARTpool siRNA targeting LINC00941 (si941). (A) qRT‐PCR analysis of LINC00941 and selected DNA‐repair genes 48 h after transfection (*n* = 3). Data are shown as fold‐change relative to siCTRL (mean ± SD). HMBS was used as an endogenous control. Brown and blue bars indicate genes whose expression changes in PDAC in the same direction as LINC00941 (higher in tumors) or in the opposite direction (lower in tumors), respectively, in the WGCNA dataset described by da Paixão et al. [24]. Statistical testing: Paired one‐tailed (LINC00941) or two‐tailed (DNA repair genes) Student's *t*‐test for normally distributed data (Shapiro–Wilk) and two‐tailed Wilcoxon signed‐rank test for non‐normal data. Significance: *p < 0.05; **p < 0.01; ****p < 0.001. Exact *p* values and effect sizes are reported (Cohen's *d* for parametric tests; rank‐biserial correlation for nonparametric tests). (B) Single‐cell RNA‐seq enrichment dot plot for LINC00941‐correlated DNA‐repair genes comparing Ductal 1 and Ductal 2 epithelial subpopulations. Dot size indicates the percentage of cells in that subpopulation with detectable expression as indicated; dot color indicates the enrichment score as indicated. Differential expression testing was performed on normalized, log_2_‐transformed counts using the Wilcoxon rank‐sum test, and *p* values were adjusted for multiple testing (Benjamini–Hochberg), with *p*adj < 0.05 considered significant. Log_2_ fold change (log_2_FC) values are indicated for significant enrichments. n.s., not significant. (C) Alkaline comet assay measuring DNA fragmentation. At 48 h post‐transfection, cells were either left untreated (UT or not treated) or exposed to 10 Gy γ‐irradiation and collected at 15 min, 30 min, 1 h, 2 h, and 4 h after irradiation. *Left*: Representative single‐nucleus comet images (*n* = 2). *Right*: Dot plots of olive tail moment (OTM) for individual nuclei at each time point; horizontal bars indicate mean ± SD. Data derived from 50 nuclei per condition per experiment, pooled from two independent experiments (total *n* = 100 nuclei per condition per time point). Statistical analysis was performed using two‐way ANOVA with Tukey's post hoc test. Significance between groups is indicated by horizontal bars (****p < 0.001).

Since DNA repair is essential for maintaining genomic integrity and promoting tumor progression, we next assessed the effect of LINC00941 knockdown on the repair of DNA strand breaks induced by γ‐irradiation (10 Gy) using the alkaline comet assay, which detects SSBs, including alkali‐labile sites, as well as double‐strand breaks (DSBs). Control and LINC00941‐depleted cells exhibited similar levels of DNA damage 15 min after irradiation, but LINC00941‐silenced cells showed significantly higher residual DNA damage at all subsequent time points (30 min to 4 h) (Figure 5C). At 4 h, DNA damage in control cells had returned to baseline levels, whereas LINC00941‐depleted cells still retained substantial DNA damage. Thus, LINC00941 depletion delays the resolution of radiation‐induced DNA strand breaks.

### 3.6 LINC00941 Contributes to Gemcitabine Chemoresistance

Since enhanced DNA repair is often associated with chemoresistance [37], we hypothesized that LINC00941 promotes resistance to gemcitabine, a nucleoside analog routinely used in PDAC therapy, both for advanced PDAC as well as an adjuvant therapy for patients eligible for surgical resection. To test this, we silenced LINC00941 in AsPC‐1 cells and assessed cell death following treatment with 10, 20, and 100 μM gemcitabine for 5 days (Figure 6A). These concentrations were chosen because prior pharmacokinetic studies in pancreatic cancer patients using fixed‐dose rate infusions have reported plasma gemcitabine concentrations of ~15 μM. Therefore, our in vitro doses of 10–20 μM are within the clinically relevant range [38], with 100 μM representing a suprapharmacological dose.

**FIGURE 6:**
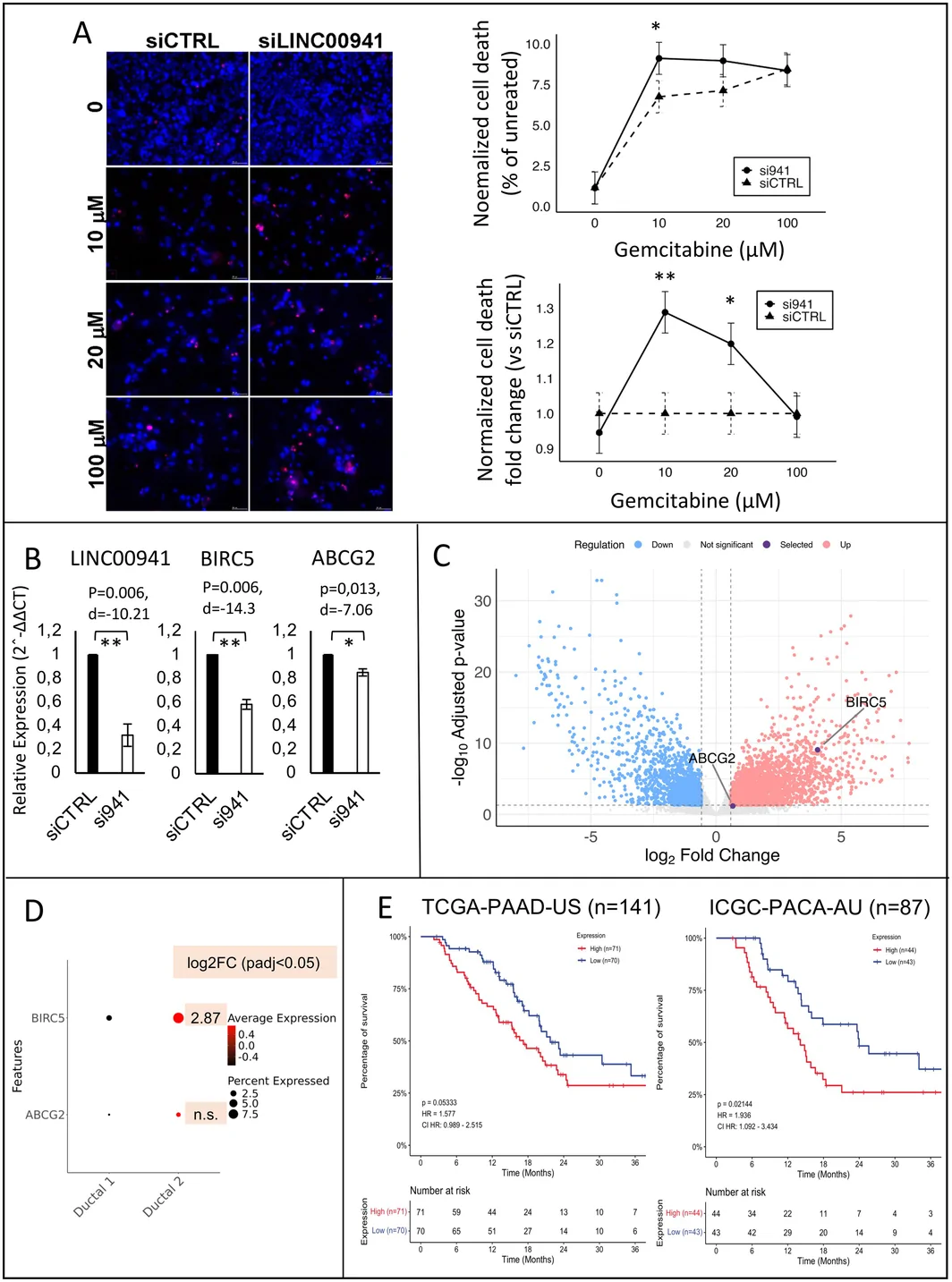
LINC00941 depletion sensitizes AsPC‐1 cells to gemcitabine. AsPC‐1 cells were transfected with a nontargeting control siRNA (siCtrl) or with a siRNA SMARTpool targeting LINC00941 (siLINC00941) as described in Section 2. After 48 h, the cells were treated with the indicated Gemcitabine doses for 5 days, and cell death was analyzed by Hoechst/PI staining. (A) *Left*: Representative fluorescence images (Hoechst, blue; PI, red) from three independent experiments. *Right*: Normalized cell death graphs as mean ± SD (*n* = 3). *Upper*: Graph represents percent dead values normalized to the 0 μM dose within each siRNA to visualize the overall gemcitabine dose–response. *Lower*: Graph represents percent dead cell values normalized to the siCTRL group at each gemcitabine concentration to highlight siRNA effects independently of gemcitabine dose. Two‐way aligned rank transform (ART) ANOVA, a nonparametric factorial test, detected significant main effects of siRNA (*F*_1,14_ = 10.45, *p* = 0.006), gemcitabine dose (*F*_3,14_ = 6.73, *p* = 0.005), and a significant siRNA × dose interaction (*F*_3,14_ = 7.25, *p* = 0.004). Post hoc contrasts (emmeans, Tukey‐adjusted) were used for pairwise comparisons in both graphs (*p < 0.05, **p < 0.01). (B) qRT‐PCR analysis of LINC00941 and selected chemoresistance‐related genes 48 h after transfection (*n* = 3). Data are shown as fold change relative to siCTRL (mean ± SD). HMBS was used as an endogenous control for LINC00941 and BIRC5, and GAPDH was used as an endogenous control for ABCG2. Statistical significance was assessed using paired one‐tailed (LINC00941) or two‐tailed (ABCG2 and BIRC5) Student's *t*‐test. Significance levels are denoted as *p < 0.05, **p < 0.01. Exact *p* values and Cohen's *d* effect sizes are indicated. (C) Volcano plot of protein‐coding gene expression changes in paired PDAC tumors and adjacent pancreatic tissue (APT, *n* = 14). The *x*‐axis represents log_2_ fold change (log_2_FC, PDAC vs. APT), and the *y*‐axis represents –log_10_ adjusted *p* value. Dotted lines indicate the thresholds for significance (log_2_FC ≥ |0.58|, adjusted *p* < 0.05). Genes are colored by significance status (upregulated, downregulated, not significant), with ABCG2 and BIRC5 highlighted. (D) Single‐cell RNA‐seq enrichment dot plot for ABCG2 and BIRC5 comparing Ductal 1 and Ductal 2 epithelial subpopulations. Dot size indicates the percentage of cells in that subpopulation with detectable expression, and dot color indicates the enrichment score. Differential expression testing was performed on normalized, log_2_‐transformed counts using the Wilcoxon rank‐sum test, and *p* values were adjusted for multiple testing (Benjamini–Hochberg), with *p*adj < 0.05 considered significant. Log_2_FC values are indicated. n.s., not significant. (E) Kaplan–Meier survival analysis based on BIRC5 expression in the TCGA (PAAD‐US, *n* = 141) and ICGC (PACA‐AU, *n* = 87) cohorts. Patients were stratified into high‐ and low‐expression groups using the median expression cutoff. Differences in survival were evaluated using the log‐rank test; log‐rank *p* values and hazard ratios (HRs) with 95% confidence intervals are shown.

When cell death was normalized to the 0 μM dose, LINC00941 depletion significantly increased cell death at 10 μM gemcitabine by 27.5% (*p* = 0.047), whereas the increase at 20 μM was not statistically significant (*p* = 0.12) (Figure 6A, upper graph). Importantly, when the data were normalized to the siCTRL condition at each dose, the sensitizing effect of LINC00941 knockdown was even more evident: cell death was significantly higher at both 10 μM (*p* = 0.0039) and 20 μM (*p* = 0.032) (Figure 6A, lower graph). At 100 μM, however, gemcitabine‐induced cytotoxicity dominated, masking additional contributions from LINC00941. These results demonstrate that LINC00941 depletion sensitizes PDAC cells to gemcitabine, with the strongest effect observed at clinically relevant doses, thereby supporting a role for this lncRNA in promoting chemoresistance.

Additionally, LINC00941 knockdown reduced expression of chemoresistance‐related genes ABCG2 and BIRC5 by 15% and 42%, respectively (Figure 6B). ABCG2 is a key drug efflux transporter implicated in PDAC chemoresistance [39], and BIRC5, also called Survivin, is a member of the inhibitor of apoptosis (IAP) family and has also been found to promote PDAC resistance to gemcitabine [40]. In tumor versus APT, both genes exhibit log_2_FC values above the differential expression threshold (log_2_FC > |0.58|), although only BIRC5 reached statistical significance (*p*adj = 8.37E−10), showing a marked fold change increase (Figure 6C). ABCG2 enrichment in Type 2 ductal cells was not statistically significant. Both are enriched in Type 2 ductal cells in single‐cell analysis, with BIRC5 displaying a significant fold change increase in Type 2 ductal cells (log_2_FC = 2.87, *p*adj = 2.89E−34; Figure 6D). Finally, high BIRC5 expression correlates with reduced overall survival in PDAC patients from both the TCGA and ICGC cohorts (Figure 6E). These results indicate that LINC00941 contributes to PDAC chemoresistance, potentially by enhancing DNA repair, promoting drug efflux mechanisms, and inhibiting chemotherapy‐induced apoptosis.

### 3.7 LINC00941‐Correlated Gene Signatures Reinforce Its Role in PDAC Aggressiveness and Chemoresistance

Finally, to gain further insight into the role of LINC00941 in KRAS‐mutant PDAC, we performed co‐expression analysis using the TCGA cohort. For downstream analyses, we applied a cutoff of Spearman correlation coefficient ≥ 0.65, yielding 393 highly co‐expressed genes with LINC00941 (Supplementary Table 3). Among these, 13 were also differentially expressed in KRAS‐mutant PDAC patients (Figure 7A). Consistent with the importance of LINC00941 in KRAS‐driven PDAC, single‐cell analysis showed that most of these LINC00941‐correlated DEGs were enriched in Type 2 ductal cells compared with Type 1 ductal cells (Figure 7A).

**FIGURE 7:**
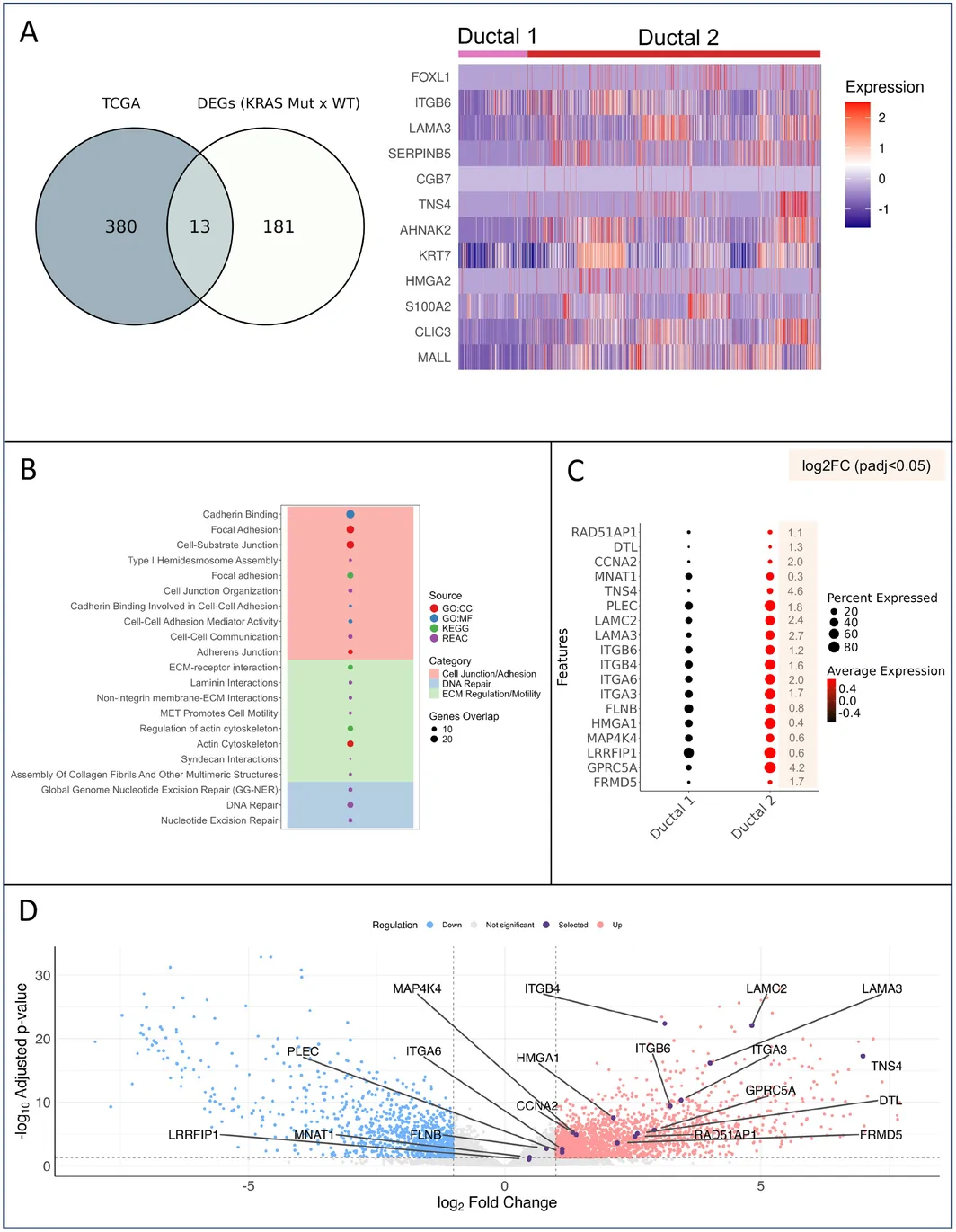
LINC00941 correlated genes are enriched in pathways related to ECM remodeling and DNA repair. (A) *Left*: Overlap between genes significantly correlated with LINC00941 in the TCGA cohort (Spearman's *ρ* ≥ 0.65) and genes differentially expressed by KRAS mutation status in the same cohort. *Right*: Heatmap of the overlapping genes in single‐cell RNA‐seq data, comparing Ductal 1 (pink bar) and Ductal 2 (red bar) epithelial populations. *Z*‐score‐normalized expression values are shown. (B) Functional enrichment analysis of the top 300 LINC00941‐correlated genes. Dot plot of significantly enriched pathways grouped into functional categories (Cell junction/Adhesion, ECM regulation/Motility, and DNA repair, as indicated). Dot size indicates the number of LINC00941‐correlated genes in each pathway (Genes Overlap); dot color denotes the functional database used for annotation (Source). (C) Single‐cell RNA‐seq enrichment dot plot of representative genes from the pathways in (B), comparing Ductal 1 and Ductal 2 epithelial populations. Dot size indicates the percentage of cells in that subpopulation with detectable expression, and dot color indicates the enrichment score. Differential expression was tested on normalized log_2_‐transformed counts using the Wilcoxon rank‐sum test, with multiple testing correction (Benjamini–Hochberg), and *p*adj < 0.05 was considered significant. Log_2_FC values are indicated. (D) Volcano plot of protein‐coding gene expression in paired PDAC tumors versus adjacent pancreatic tissue (APT, *n* = 14). The *x*‐axis shows log_2_ fold change (log_2_FC, PDAC vs. APT), and the *y*‐axis shows –log_10_ adjusted *p* value. Dotted lines indicate the thresholds for significance (log_2_FC ≥ |0.58|, adjusted *p* < 0.05). Genes are colored by significance status (upregulated, downregulated, not significant), and LINC00941‐correlated genes in enriched pathways are highlighted.

Functional enrichment of the top 300 LINC00941‐correlated genes revealed significant associations with DNA repair, cell motility, extracellular matrix regulation, and cell adhesion pathways (Supplementary Table 4 and Figure 7B). Representative genes from these categories were not only enriched in Type 2 ductal cells (Figure 7C) but also significantly upregulated in PDAC tumors (15 out of 18; Figure 7D). Importantly, ITGA3 and RAD51AP1 were also significantly associated with patient survival (Supplementary Figure 3). Together, these findings reinforce our experimental results and support a model in which LINC00941 promotes tumor aggressiveness and contributes to chemoresistance and poor prognosis in KRAS‐driven PDAC. In summary, our integrative analyses establish LINC00941 as a KRAS‐regulated lncRNA with distinct functional relevance in PDAC. We show that LINC00941 is consistently upregulated in KRAS‐mutant tumors, enriched in malignant epithelial cells, and associated with poor patient prognosis. Functionally, LINC00941 drives an aggressive phenotype characterized by enhanced migration, invasion, and efficient DNA repair, while also conferring resistance to gemcitabine. These observations are supported by transcriptomic data linking LINC00941 to gene networks controlling the DDR, cell motility, and extracellular matrix regulation. Collectively, these findings position LINC00941 as both a biomarker of PDAC aggressiveness and a potential therapeutic target for overcoming KRAS‐driven tumor progression and treatment resistance.

## 4 Discussion

Despite decades of research, PDAC remains a major public health challenge. It is still difficult to detect and has an extremely low survival rate, even with advancements in medicine [3]. KRAS mutations are found in over 90% of cases, playing a key role in oncogenic transformation [41, 42]. While efforts to directly inhibit KRAS have been made, and mutation‐specific inhibitors for KRAS^G12C^ have generated hope in lung cancer, this approach still faces significant hurdles in PDAC and other cancers, where G12C mutations are rare, and resistance mechanisms are complex [4, 6]. Therefore, deciphering the molecular pathways activated by oncogenic KRAS in PDAC is essential to identify potential new therapeutic targets.

The role of lncRNAs in orchestrating the malignant phenotype is becoming increasingly evident. These molecules have been implicated in all cancer hallmarks, with growing evidence of their significance in PDAC [43]. Although the interaction between lncRNAs and KRAS has been studied, findings in this area remain incomplete. Some studies describe lncRNAs that regulate KRAS expression, often through miRNA sequestration [44]. However, reports of lncRNAs acting as downstream effectors of KRAS are scarce, especially in PDAC [45, 46, 47, 48, 49, 50, 51]. This study addresses this gap by establishing the KRAS/LINC00941 axis as a pathway with significant clinical and functional relevance in PDAC.

To identify KRAS‐regulated lncRNAs, we conducted a differential expression analysis of the TCGA PDAC dataset stratified by KRAS mutation status and identified 49 differentially expressed lincRNAs. Although few KRAS‐regulated lncRNAs in PDAC have been characterized, our analysis identified *UCA1*, a known oncogenic lncRNA previously shown to be upregulated by KRAS in PDAC [48, 49]. Additionally, we found that the expression of known PDAC‐associated oncogenic lncRNAs, such as LINC01559 [52], LINC01133 [53], LINC00941 [54], and LINC00462 [55], was significantly associated with KRAS status. These findings suggest that these lncRNAs likely function as downstream targets of KRAS signaling in pancreatic cancer.

In contrast, the KRAS‐associated lncRNAs identified in this study were not differentially expressed according to KRAS mutation status in a TCGA cohort of colorectal cancer patients [45]. This observation underscores the context‐dependent nature of lncRNA expression and function and supports the notion that the lncRNAs identified here may act as PDAC‐specific KRAS biomarkers.

Among the top candidates, LINC00941 was positively regulated by KRAS in both gain‐ and loss‐of‐function models in pancreatic cells, underscoring its potential as a bona fide KRAS effector. LINC00941 has been implicated in various cancers, including PDAC [56, 57]. In a previous study, we identified LINC00941 as upregulated in PDAC [24]. Here, we confirmed that LINC00941 expression is associated with poor survival in both the TCGA and ICGC PDAC cohorts and demonstrated its enrichment in the malignant epithelial cell population, indicating a cancer cell‐intrinsic function. Functionally, LINC00941 silencing reduced PDAC cell migration and invasion, two key traits of malignant behavior, while having no evident effect on cell viability or death. These findings align with recent reports linking *LINC00941* to worse prognosis and the regulation of malignant traits in PDAC [58, 59, 60, 61].

We had previously identified that LINC00941 was co‐expressed with DNA repair genes in PDAC [24]. We now show that LINC00941 depletion delays the resolution of radiation‐induced DNA strand breaks and reduces the expression of genes involved in DNA repair. Among the most consistently regulated targets were PPP4C, a phosphatase that facilitates HR via PLK1 dephosphorylation [62], and BACH1, a transcription regulator that enhances RAD51‐mediated HR repair and cell cycle progression [63, 64]. Notably, both are significantly enriched in malignant Type 2 ductal cells. BACH1 expression is also increased in KRAS mutant patients, and PPP4C is associated with cancer progression and poor prognosis in other tumor types [65].

In LINC00941‐depleted cells, residual DNA damage diverged from that of control cells as early as 30 min after irradiation. These kinetics resemble those reported for SSB repair‐deficient cells, such as XRCC1‐deficient cells, whereas cells with severe DSB repair defects, such as XRCC4‐deficient cells, show only minor differences in the alkaline comet assay during the first hour after irradiation [66]. Therefore, although a contribution of DSB repair at later time points cannot be excluded, our data suggest that LINC00941 depletion predominantly affects SSB repair. Consistent with this possibility, the DNA repair co‐expression module containing LINC00941 also includes SSB repair and base excision repair genes, such as POLB, PARP2, and MBD4. Importantly, in proliferating cells, unrepaired SSBs can be converted into DSBs when encountered by replication forks, and these replication‐associated DSBs are predominantly repaired by HR [67, 68, 69]. Thus, the reduced expression of the HR‐associated factors PPP4C and BACH1 could further compromise the resolution of DNA damage in LINC00941‐depleted cells, particularly at later time points, and may also contribute to their increased sensitivity to gemcitabine, which induces replication stress. These possibilities remain to be tested.

LINC00941 knockdown also reduced the expression of TOP2A, which was strongly enriched in malignant Type 2 ductal cells. TOP2A resolves DNA topological constraints during replication and mitosis and has been implicated in the DDR [70]. However, because TOP2A expression is tightly linked to cell proliferation and its downregulation was not observed in PANC‐1 cells, this effect may reflect changes in cell cycle distribution rather than a direct role of LINC00941 in DNA repair.

Enhanced DNA repair capacity is a prominent mechanism contributing to chemoresistance [37]. Consistently, we found that LINC00941 also promotes gemcitabine chemoresistance. This finding is in agreement with emerging evidence that other noncoding RNAs can enhance gemcitabine tolerance through the activation of DNA repair pathways [71, 72].

Beyond DNA repair, our findings suggest that LINC00941 contributes to PDAC chemoresistance by regulating downstream effectors involved in drug efflux and apoptotic resistance. LINC00941 knockdown reduced the expression of ABCG2, an ATP‐binding cassette transporter that mediates efflux of several molecules and is implicated in multidrug resistance and tumor progression [73], as well as BIRC5 (Survivin), a member of the IAP family [74]. BIRC5, but not ABCG2, was significantly upregulated in PDAC bulk tumors and enriched in malignant PDAC cells, and BIRC5 expression was strongly associated with reduced overall survival in PDAC patients.

The relevance of BIRC5 is supported by previous studies demonstrating its role in promoting resistance to gemcitabine in PDAC models [40, 75, 76]. Mechanistically, BIRC5 inhibits caspase‐dependent apoptosis and facilitates cell cycle progression, thereby allowing cancer cells to evade chemotherapy‐induced cell death [74]. Interestingly, BIRC5 has also been linked to enhanced DNA damage repair and mitotic regulation, further reinforcing its role in sustaining tumor cell survival under genotoxic stress [77, 78]. Finally, high BIRC5 expression has been consistently reported across solid tumors as a marker of aggressive disease and poor prognosis [74], which is in agreement with our survival analyses in PDAC. Thus, given the stronger upregulation of BIRC5 in comparison to ABCG2, its significant prognostic association, and its known capacity to suppress apoptosis, it is likely that LINC00941‐driven chemoresistance is mediated largely through apoptosis inhibition via BIRC5, complementing the effects on drug efflux and DNA repair. LINC00941‐mediated upregulation of ABCG2 and BIRC5 correlates with the aggressive and chemoresistant phenotype frequently observed in PDAC tumors.

Furthermore, pathway enrichment analysis of LINC00941‐correlated genes supports a model in which LINC00941 promotes PDAC aggressiveness and chemoresistance via two major axes: (1) enhancing invasive/motile behavior through ECM and adhesion dynamics, and (2) increasing the capacity to repair DNA strand breaks and survive DNA‐targeting therapies. Driving these enrichments are genes like ITGA3 and RAD51AP1, both upregulated in PDAC and linked to poor survival. ITGA3, an integrin alpha subunit involved in cell–ECM adhesion and signaling, has been linked to invasion, metastasis, and poor prognosis, with functional studies showing that its inhibition reduces PDAC growth and invasiveness [79, 80]. RAD51AP1, a RAD51‐interacting protein that promotes HR, is likewise upregulated in PDAC and associated with poor survival, likely by enhancing DNA repair capacity and conferring resistance to genotoxic therapies [81, 82]. Together, these data illustrate how LINC00941‐associated networks converge on ECM remodeling and genomic stability to fuel an aggressive, therapy‐resistant phenotype.

This study has limitations. First, KRAS wild‐type PDAC is rare, and the small number of wild‐type samples in public cohorts (*n* = 14 in TCGA; *n* = 9 in ICGC) limits statistical power. This, together with the use of total RNA libraries in ICGC, may explain why the TCGA differences did not reach significance in that cohort. Second, our internal validation set was small (seven PDAC and five APT samples), with KRAS status available for only five tumors. This precluded analysis according to KRAS status. Third, the increase in LINC00941 induced by oncogenic KRAS in HPDE cells did not reach significance (*p* = 0.07), and functional experiments relied mainly on siRNA‐mediated knockdown in AsPC‐1 cells. Fourth, the type of DNA lesion whose repair depends on LINC00941 should be confirmed with lesion‐specific assays. Finally, the contribution of the KRAS/LINC00941 axis to DNA repair and chemoresistance remains to be established in in vivo models.

In conclusion, our study establishes LINC00941 as a key KRAS‐regulated lncRNA that drives PDAC aggressiveness by enhancing migration, invasion, efficient repair of DNA strand breaks, and chemoresistance. LINC00941 also sustains the expression of DNA repair factors, such as PPP4C and BACH1, and of chemoresistance mediators, including ABCG2 and BIRC5. Among these, BIRC5 stands out as a strong predictor of poor survival and a likely major effector of LINC00941‐mediated resistance to gemcitabine. The integration of these validated targets with pathway analyses strengthens the model that LINC00941 promotes tumor progression through both apoptosis inhibition and efficient DNA strand break repair. These findings highlight the KRAS/LINC00941 axis as a clinically relevant signaling node in PDAC and suggest that therapeutic strategies aimed at targeting LINC00941 or its key effectors could offer the dual benefit of limiting metastasis and sensitizing PDAC tumors to chemotherapy.

## Funding

This work was supported by the São Paulo Research Foundation (FAPESP) (2016/19757‐2, 2022/06092‐3, 2016/11639‐0, 2024/21592‐8), the National Council for Scientific and Technological Development (CNPq) (306828/2019‐7, 306778/2022‐0, 140042/2017‐2), the Associazione Italiana per la Ricerca sul Cancro (AIRC) (25734), and the Brazilian Federal Agency for Support and Evaluation of Graduate Education (CAPES) (Finance Code 001).

## Conflicts of Interest

The authors declare no conflicts of interest.

## Supporting information


**Supplementary Figure 1** Expression of selected lincRNAs according to KRAS mutation status in the ICGC PDAC cohort. Dot plots comparing log_2_‐transformed CPM‐normalized counts of the indicated lincRNAs in KRAS^MUT^ (*n* = 9) and KRAS^WT^ (*n* = 89) tumors in the ICGC PDAC cohort (PACA‐AU, *n* = 98). Statistical significance was assessed using two‐tailed Wilcoxon rank‐sum tests, with *p* < 0.05 considered significant.

**Supplementary Figure 2** LINC00941 depletion affects the expression of LINC00941‐correlated DNA repair genes in PANC‐1 cells. PANC‐1 cells were transfected with nontargeting control siRNA (siCTRL) or a SMARTpool siRNA targeting LINC00941 (si941). (A) qRT‐PCR analysis of LINC00941 and selected DNA‐repair genes 48 h after transfection (*n* = 3). Data are shown as fold‐change relative to siCTRL (mean ± SD). Colored bars indicate genes whose expression changes in PDAC in the same direction as LINC00941 (brown, higher in tumors) or in the opposite direction (blue, lower in tumors) in the WGCNA dataset described by da Paixão et al. [24]. Statistical significance was assessed using paired one‐tailed (LINC00941) or two‐tailed (DNA repair genes) Student's *t*‐test. Significance levels are denoted as *p < 0.05, **p < 0.01. Exact *p* values and Cohen's *d* effect sizes are indicated.

**Supplementary Figure 3** High expression of LINC00941‐correlated genes in the TCGA cohort, ITGA3 and RAD51AP1, is associated with poor survival in PDAC. Kaplan–Meier survival analysis based on ITGA3 (left) and RAD51AP1 (right) expression in the TCGA (PAAD‐US, *n* = 145) and ICGC (PACA‐AU, *n* = 87) cohorts, as indicated. Patients were stratified into high‐ and low‐expression groups using the median expression cutoff. Differences in survival were evaluated using the log‐rank test; log‐rank *p* values and hazard ratios (HRs) with 95% confidence intervals are shown.

**Supplementary Table 1** Primers used for qRT‐PCR.

**Supplementary Table 2** Differentially expressed lincRNAs in KRASmut patients (*n* = 131) versus KRAS wild‐type patients (*n* = 14) in the PAAD‐US TCGA dataset.

**Supplementary Table 3** LINC00941 TCGA significant spearman correlations.

**Supplementary Table 4** TCGA top 300 LINC00941‐correlated genes enrichment—Significant terms.

**Supplementary Table 5** KRAS alterations in the TCGA PDAC cohort (PAAD‐US) used for the differential expression analysis.

**Supplementary Table 6** Clinicopathological characteristics and KRAS mutational status of the internal validation set (qRT‐PCR; Figure 3A).

**Supplementary Table 7** Key resources and research resource identifiers (RRIDs).

## Data Availability

The underlying differential expression data, correlation results, and pathway enrichment data (Supplementary Tables [Link], [Link]) have been archived in the Zenodo public data repository under the following persistent identifier: https://doi.org/10.5281/zenodo.17661947. All other datasets generated during and/or analyzed during the current study are available from the corresponding author on reasonable request.
