# Supplementary material for: Long Noncoding RNA LINC00941 Links Oncogenic KRAS Signaling to Aggressiveness, DNA Repair, and Chemoresistance in Pancreatic Cancer
Source: IUBMB Life. 2026 Nov 9;78(11):e70140. doi: 10.1002/iub.70140 (PMC13748920; doi:10.1002/iub.70140)
Supplement: Supplementary file 1 [file IUB-78-0-s002.pdf]

Supplementary Table 5. KRAS alterations in the TCGA PDAC cohort (PAAD-US) used for the differential expression analysis.

A. Distribution of KRAS alterations among KRAS-mutant tumors

KRAS alteration	n	% of KRAS-mutant tumors (n = 131)
G12D	58	44.3
G12V	38	29.0
G12R	25	19.1
Q61H	6	4.6
Q61R	2	1.5
G13C	1	0.8
G12C	1	0.8
**Total**	**131**	**100.0**

Total cohort: n = 145 (131 KRAS-mutant and 14 KRAS wild-type tumors).

KRAS mutation calls were retrieved from cBioPortal (Pancreatic Adenocarcinoma, TCGA Firehose Legacy; whole-exome sequencing).

Tumors harboring more than one KRAS mutation were classified according to the alteration with the highest variant allele frequency (VAF): TCGA-2J-AAB1 (G12R, 0.13; G12D, 0.04), TCGA-2J-AABH (G12V, 0.19; G12S, 0.19), TCGA-FB-A78T (G13C, 0.15; G12A, 0.14) and TCGA-YB-A89D (G12R, 0.17; G12D, 0.01).

B. KRAS alterations per patient

Patient ID	KRAS alteration (predominant)	All KRAS alterations detected (VAF)
TCGA-2J-AAB1	G12R	G12R (0.13); G12D (0.04)
TCGA-2J-AAB4	G12V	G12V (0.10)
TCGA-2J-AAB6	G12V	G12V (0.53)
TCGA-2J-AAB8	G12R	G12R (0.09)
TCGA-2J-AAB9	G12D	G12D (0.04)
TCGA-2J-AABE	G12R	G12R (0.15)
TCGA-2J-AABF	G12D	G12D (0.10)
TCGA-2J-AABH	G12V	G12V (0.19); G12S (0.19)
TCGA-2J-AABK	G12V	G12V (0.08)
TCGA-2J-AABO	G12V	G12V (0.28)
TCGA-2J-AABR	G12V	G12V (0.04)
TCGA-2J-AABU	G12R	G12R (0.35)
TCGA-2J-AABV	G12V	G12V (0.05)
TCGA-2L-AAQA	G12V	G12V (0.30)
TCGA-2L-AAQE	G12D	G12D (0.29)
TCGA-2L-AAQI	G12V	G12V (0.11)
TCGA-2L-AAQJ	G12D	G12D (0.23)
TCGA-2L-AAQL	Q61R	Q61R (0.19)
TCGA-3A-A9I7	G12D	G12D (0.07)
TCGA-3A-A9I9	G12R	G12R (0.11)
TCGA-3A-A9IB	G12D	G12D (0.21)
TCGA-3A-A9IC	G12R	G12R (0.18)
TCGA-3A-A9IH	G12R	G12R (0.16)
TCGA-3A-A9IU	G12D	G12D (0.24)
TCGA-3A-A9IX	G12D	G12D (0.03)
TCGA-3A-A9IZ	G12D	G12D (0.27)
TCGA-3E-AAAY	G12D	G12D (0.03)
TCGA-3E-AAAZ	G12V	G12V (0.15)

Patient ID	KRAS alteration (predominant)	All KRAS alterations detected (VAF)
TCGA-F2-6879	G12V	G12V (0.45)
TCGA-F2-A44G	G12D	G12D (0.15)
TCGA-F2-A7TX	G12D	G12D (0.16)
TCGA-F2-A8YN	G12R	G12R (0.35)
TCGA-FB-A4P5	G12D	G12D (0.02)
TCGA-FB-A545	G12V	G12V (0.36)
TCGA-FB-A5VM	Q61H	Q61H (0.13)
TCGA-FB-A78T	G13C	G13C (0.15); G12A (0.14)
TCGA-FB-AAPS	G12D	G12D (0.05)
TCGA-FB-AAPU	G12R	G12R (0.35)
TCGA-FB-AAPY	G12D	G12D (0.10)
TCGA-FB-AAPZ	G12R	G12R (0.34)
TCGA-FB-AAQ0	G12R	G12R (0.27)
TCGA-FB-AAQ1	G12D	G12D (0.20)
TCGA-FB-AAQ2	G12D	G12D (0.30)
TCGA-FB-AAQ3	G12D	G12D (0.14)
TCGA-H6-8124	G12V	G12V (0.69)
TCGA-H6-A45N	G12V	G12V (0.05)
TCGA-HV-A5A3	G12D	G12D (0.51)
TCGA-HV-A5A4	G12V	G12V (0.46)
TCGA-HV-A5A5	G12D	G12D (0.07)
TCGA-HV-A5A6	G12D	G12D (0.37)
TCGA-HV-A7OL	G12D	G12D (0.24)
TCGA-HV-AA8V	G12V	G12V (0.09)
TCGA-HV-AA8X	G12D	G12D (0.24)
TCGA-HZ-7919	G12R	G12R (0.31)
TCGA-HZ-7922	Q61H	Q61H (0.39)
TCGA-HZ-7925	G12V	G12V (0.11)
TCGA-HZ-7926	G12D	G12D (0.12)
TCGA-HZ-8001	G12V	G12V (0.07)
TCGA-HZ-8002	G12D	G12D (0.07)
TCGA-HZ-8003	G12R	G12R (0.04)
TCGA-HZ-8005	Q61H	Q61H (0.27)
TCGA-HZ-8315	G12D	G12D (0.13)
TCGA-HZ-8519	G12D	G12D (0.05)
TCGA-HZ-8636	G12V	G12V (0.28)
TCGA-HZ-8637	G12V	G12V (0.11)
TCGA-HZ-A49G	G12D	G12D (0.08)
TCGA-HZ-A49H	G12V	G12V (0.13)
TCGA-HZ-A49I	G12D	G12D (0.30)
TCGA-HZ-A4BH	G12D	G12D (0.19)
TCGA-HZ-A4BK	G12D	G12D (0.31)
TCGA-HZ-A77O	G12V	G12V (0.20)
TCGA-HZ-A77P	G12V	G12V (0.07)
TCGA-HZ-A77Q	G12R	G12R (0.07)
TCGA-HZ-A8P0	Q61H	Q61H (0.20)

Patient ID	KRAS alteration (predominant)	All KRAS alterations detected (VAF)
TCGA-HZ-A8P1	G12D	G12D (0.29)
TCGA-HZ-A9TJ	G12V	G12V (0.25)
TCGA-IB-7644	G12V	G12V (0.16)
TCGA-IB-7645	G12V	G12V (0.11)
TCGA-IB-7646	G12D	G12D (0.21)
TCGA-IB-7647	G12V	G12V (0.09)
TCGA-IB-7649	Q61H	Q61H (0.09)
TCGA-IB-7651	G12D	G12D (0.13)
TCGA-IB-7652	G12R	G12R (0.09)
TCGA-IB-7885	G12V	G12V (0.07)
TCGA-IB-7886	G12C	G12C (0.70)
TCGA-IB-7887	G12R	G12R (0.13)
TCGA-IB-7889	G12V	G12V (0.15)
TCGA-IB-7890	Q61R	Q61R (0.30)
TCGA-IB-7893	G12D	G12D (0.18)
TCGA-IB-7897	G12D	G12D (0.01)
TCGA-IB-8126	G12D	G12D (VAF not available)
TCGA-IB-8127	G12D	G12D (0.41)
TCGA-IB-A5SO	G12D	G12D (0.10)
TCGA-IB-A5SP	G12D	G12D (0.58)
TCGA-IB-A5SS	G12D	G12D (0.15)
TCGA-IB-A5ST	G12D	G12D (0.04)
TCGA-IB-A6UF	G12R	G12R (0.37)
TCGA-IB-A6UG	G12D	G12D (0.10)
TCGA-IB-A7LX	G12D	G12D (0.51)
TCGA-IB-A7M4	G12D	G12D (0.22)
TCGA-IB-AAUM	G12D	G12D (0.06)
TCGA-IB-AAUN	G12D	G12D (0.26)
TCGA-IB-AAUO	G12D	G12D (0.18)
TCGA-IB-AAUP	G12D	G12D (0.04)
TCGA-IB-AAUQ	G12D	G12D (0.11)
TCGA-IB-AAUR	G12R	G12R (0.04)
TCGA-IB-AAUS	G12D	G12D (0.02)
TCGA-IB-AAUU	G12R	G12R (0.23)
TCGA-LB-A9Q5	G12D	G12D (0.05)
TCGA-M8-A5N4	G12D	G12D (0.08)
TCGA-OE-A75W	G12D	G12D (0.32)
TCGA-PZ-A5RE	Q61H	Q61H (0.42)
TCGA-Q3-A5QY	G12V	G12V (0.01)
TCGA-Q3-AA2A	G12V	G12V (0.31)
TCGA-RB-A7B8	G12R	G12R (0.10)
TCGA-RB-AA9M	G12V	G12V (0.05)
TCGA-S4-A8RM	G12V	G12V (0.26)
TCGA-S4-A8RO	G12D	G12D (0.31)
TCGA-S4-A8RP	G12R	G12R (0.22)
TCGA-US-A774	G12D	G12D (0.05)

**Patient ID**	**KRAS alteration (predominant)**	**All KRAS alterations detected (VAF)**
TCGA-US-A779	G12D	G12D (0.26)
TCGA-US-A77G	G12R	G12R (0.17)
TCGA-XD-AAUG	G12V	G12V (0.04)
TCGA-XD-AAUI	G12V	G12V (0.12)
TCGA-XD-AAUL	G12R	G12R (0.11)
TCGA-XN-A8T3	G12D	G12D (0.21)
TCGA-XN-A8T5	G12R	G12R (0.04)
TCGA-YB-A89D	G12R	G12R (0.17); G12D (0.01)
TCGA-YH-A8SY	G12V	G12V (0.22)
TCGA-YY-A8LH	G12V	G12V (0.29)
TCGA-Z5-AAPL	G12V	G12V (0.02)

VAF, variant allele frequency in the tumor sample.
