# Supplementary material for: Long Noncoding RNA LINC00941 Links Oncogenic KRAS Signaling to Aggressiveness, DNA Repair, and Chemoresistance in Pancreatic Cancer
Source: IUBMB Life. 2026 Nov 9;78(11):e70140. doi: 10.1002/iub.70140 (PMC13748920; doi:10.1002/iub.70140)
Supplement: Supplementary file 2 [file IUB-78-0-s004.pdf]

SUPPLEMENTARY FIGURE 2

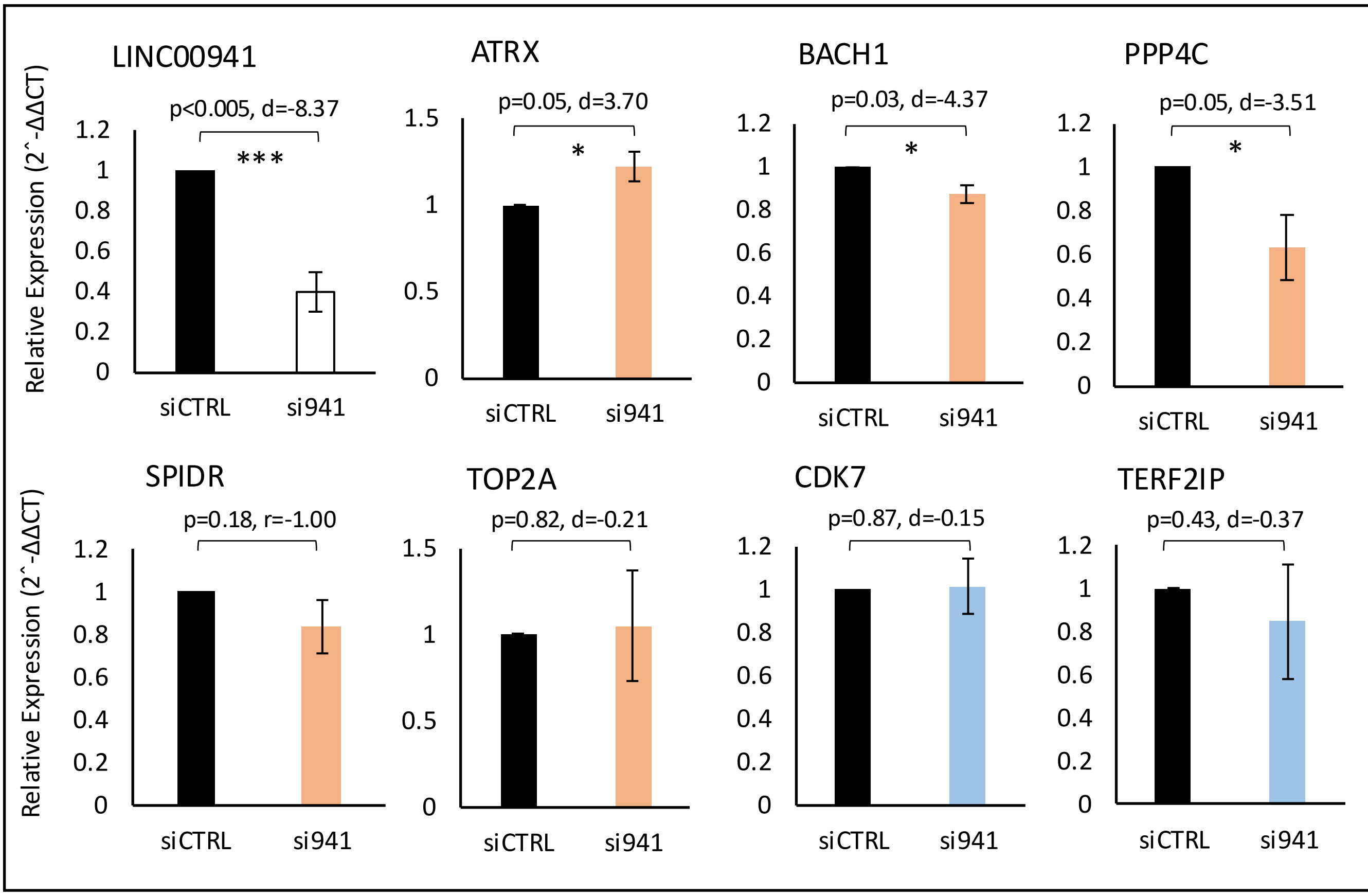

LINC00941 depletion affects the expression of LINC00941-correlated DNA repair genes in PANC-1 cells. PANC-1 cells were transfected with non-targeting control siRNA (siCTRL) or a SMARTpool siRNA targeting LINC00941 (si941). **(A)** qRT-PCR analysis of LINC00941 and selected DNA-repair genes 48 h after transfection (n = 3). Data are shown as fold-change relative to siCTRL (mean ± SD). Colored bars indicate genes whose expression changes in PDAC in the same direction as LINC00941 (brown, higher in tumors) or in the opposite direction (blue, lower in tumors) in the WGCNA dataset described by da Paixão et al (24). Statistical significance was assessed using paired one-tailed (LINC00941) or two-tailed (DNA repair genes) Student's *t*-test. Significance levels are denoted as *p < 0.05, **p < 0.01. Exact *p*-values and Cohen's *d* effect sizes are indicated.
