# Supplementary material for: Long Noncoding RNA LINC00941 Links Oncogenic KRAS Signaling to Aggressiveness, DNA Repair, and Chemoresistance in Pancreatic Cancer
Source: IUBMB Life. 2026 Nov 9;78(11):e70140. doi: 10.1002/iub.70140 (PMC13748920; doi:10.1002/iub.70140)
Supplement: Supplementary file 3 [file IUB-78-0-s005.pdf]

SUPPLEMENTARY FIGURE 1

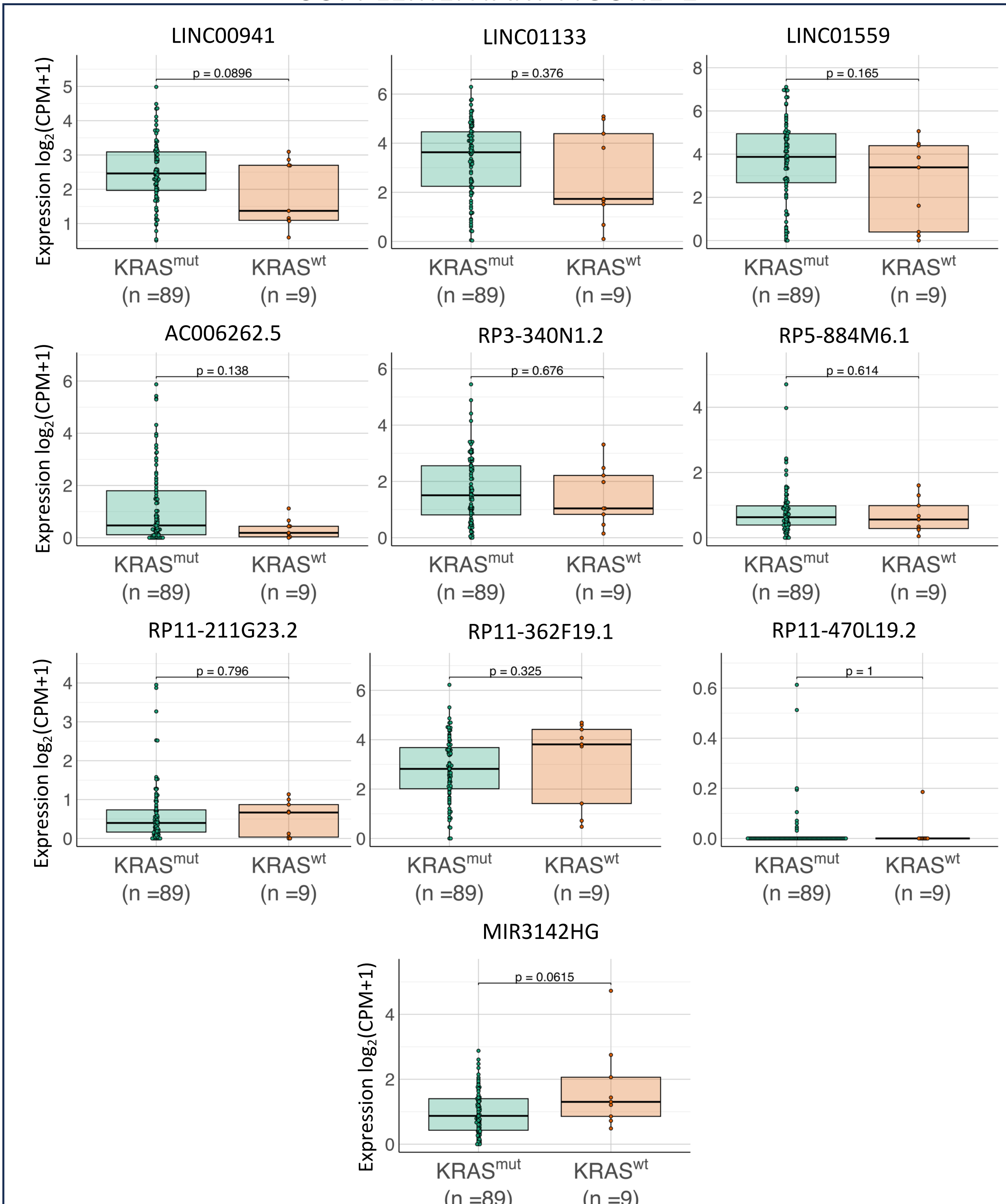

Expression of selected lincRNAs according to *KRAS* mutation status in the ICGC PDAC cohort. Dot plots comparing $\log_2$-transformed CPM-normalized counts of the indicated lincRNAs in $KRAS^{MUT}$ (n = 9) and $KRAS^{WT}$ (n = 89) tumors in the ICGC PDAC cohort (PACA-AU, n = 98). Statistical significance was assessed using two-tailed Wilcoxon rank-sum tests, with $p < 0.05$ considered significant.
