# Supplementary material for: Long Noncoding RNA LINC00941 Links Oncogenic KRAS Signaling to Aggressiveness, DNA Repair, and Chemoresistance in Pancreatic Cancer
Source: IUBMB Life. 2026 Nov 9;78(11):e70140. doi: 10.1002/iub.70140 (PMC13748920; doi:10.1002/iub.70140)
Supplement: Supplementary file 4 [file IUB-78-0-s007.pdf]

**Supplementary Table 7. Key resources and Research Resource Identifiers (RRIDs).**

| Resource type | Resource | Source | Identifier / catalog no. | RRID |
|---|---|---|---|---|
| Cell line | AsPC-1 | ATCC | CRL-1682 | RRID:CVCL_0152 |
| Cell line | PANC-1 | ATCC | CRL-1469 | RRID:CVCL_0480 |
| Cell line | HPDE6c7 | Gift from Dr. Albert S. Baldwin (UNC Chapel Hill); originally established by Ouyang et al., 2000 | — | RRID:CVCL_0P38 |
| Cell line | HPDE-KR | Gift from Dr. Albert S. Baldwin (UNC Chapel Hill); described in Qian et al., 2005 | — | Derived from RRID:CVCL_0P38 |
| Antibody | Anti-GAPDH, rabbit monoclonal (clone D16H11 XP®) | Cell Signaling Technology | 5174 | RRID:AB_10622025 |
| Antibody | Anti-α-tubulin, mouse monoclonal (clone DM1A) | Cell Signaling Technology | 3873 | RRID:AB_1904178 |
| Antibody | Anti-Pan-Ras (Ab-3), mouse monoclonal (clone RAS 10) | Calbiochem (Merck Millipore) | OP40 | RRID:AB_213400 |
| Antibody | Anti-rabbit IgG (H+L), HRP conjugate, goat polyclonal | Promega | W4011 | RRID:AB_430833 |
| Antibody | Anti-mouse IgG (H+L), HRP conjugate, goat polyclonal | Promega | W4021 | RRID:AB_430834 |
| siRNA | siGENOME SMARTpool, human KRAS | Dharmacon/Horizon Discovery | M-005069-01-0005 | — |
| siRNA | Lincode SMARTpool, LINC00941 | Dharmacon/Horizon Discovery | R-188444-00-0005 | — |
| siRNA | siGENOME Non-Targeting siRNA #3 | Dharmacon/Horizon Discovery | D-001210-03-05 | — |
| Software | R | R Foundation | — | RRID:SCR_001905 |
| Software | RSEM | Li & Dewey, 2011 | — | RRID:SCR_000262 |
| Software | DESeq2 | Bioconductor | — | RRID:SCR_015687 |
| Software | edgeR (v4.4.2) | Bioconductor | — | RRID:SCR_012802 |
| Software | Seurat (v5.0.3) | Satija Lab | — | RRID:SCR_016341 |
| Software | Enrichr (R package v3.4) | CRAN; interface to the Enrichr web server (Ma'ayan Lab) | — | RRID:SCR_001575 |

| Resource type | Resource | Source | Identifier / catalog no. | RRID |
|---|---|---|---|---|
| Software | survival (R package) | CRAN | — | RRID:SCR_021137 |
| Software | Primer3web v4.0.0 | https://primer3.ut.ee/ | — | RRID:SCR_003139 |
| Software | ImageJ | NIH | — | RRID:SCR_003070 |
| Software | StrataQuest | TissueGnostics | — | RRID:SCR_024861 |
| Software | Komet 6.0 | Andor / Oxford Instruments | — | NA |
| Database | cBioPortal | https://www.cbioportal.org/ | — | RRID:SCR_014555 |
| Database | ICGC Data Portal | https://dcc.icgc.org/ | — | RRID:SCR_021722 |
| Database | Genome Sequence Archive | CNCB-NGDC | CRA001160 | RRID:SCR_025826 |
| Database | Gene Ontology | Gene Ontology Consortium | — | RRID:SCR_002811 |
| Database | KEGG | Kanehisa Laboratories | — | RRID:SCR_012773 |
| Database | Reactome | https://reactome.org/ | — | RRID:SCR_003485 |

—, not applicable; NA, not available.
