# Supplementary material for: Long Noncoding RNA LINC00941 Links Oncogenic KRAS Signaling to Aggressiveness, DNA Repair, and Chemoresistance in Pancreatic Cancer
Source: IUBMB Life. 2026 Nov 9;78(11):e70140. doi: 10.1002/iub.70140 (PMC13748920; doi:10.1002/iub.70140)
Supplement: Supplementary file 5 [file IUB-78-0-s008.pdf]

Supplementary Table 6. Clinicopathological characteristics and KRAS mutational status of the internal validation set (qRT-PCR, Figure 3A).

Sample ID	Tissue	KRAS mutation (VAF, %)	Other variants detected	Sex	Age (years)	Histopathological classification of the resected lesion (H&E)
**Tumor samples (n = 7)**						
03T	Tumor	NA	NA	NA	NA	NA
05T	Tumor	NA	NA	NA	NA	NA
12T	Tumor	G12V (10)	—	M	81	95% PDAC, 5% inseparable intraductal neoplasia
30T	Tumor	G12R (13)	—	M	77	100% PDAC
35T	Tumor	G12D (15)	—	M	77	80% PDAC, 20% inseparable endocrine pancreatic tissue
47T	Tumor	G12R (1)	—	F	67	100% PDAC
56T	Tumor	WT	BRAF c.1799_1801del (p.V600_K601delinsE; VAF ~12%)†	F	75	100% pancreatic intraepithelial neoplasia
**Non-tumor adjacent pancreatic tissue samples (APT; n = 5)**						
14N	APT	NA	NA	NA	NA	NA
29N	APT	G12R (8)*	—	F	75	100% PDAC*
44N	APT	G12D (50)*	—	M	80	100% IPMN*
50N	APT	G12D (5)*	—	F	65	90% PDAC, 10% inseparable duodenal wall*
58N	APT	G12R (19)*	—	M	73	80% PDAC, 20% inseparable normal mucosa*

APT, adjacent pancreatic tissue; F, female; IPMN, intraductal papillary mucinous neoplasm; M, male; NA, not available; PDAC, pancreatic ductal adenocarcinoma; VAF, variant allele frequency; WT, wild-type.

Clinical data and KRAS status were not available for patients 03, 05 and 14; tumor tissue from patient 14 was not collected.

KRAS and BRAF status was determined by targeted next-generation sequencing (Illumina) of genomic DNA using a multi-gene amplicon panel covering hotspot regions including KRAS codons 12, 13, 61, 117 and 146 and BRAF codon 600, with a coverage depth of $1.8–2.5 \times 10^6$ reads at KRAS codon 12 for all sequenced samples. Low VAFs are consistent with the low neoplastic cellularity typical of PDAC.

* For APT samples, KRAS status and histopathological classification refer to the resected tumor of the same patient, not to the APT sample itself.

† In-frame BRAF deletion detected by the same sequencing panel. This deletion has been shown to activate BRAF and downstream MEK/ERK signaling (Hou P, Liu D, Xing M. Cell Cycle. 2007;6(3):377–379; Ng PKS et al. Cancer Cell. 2018;33(3):450–462.e10).
