# Supplementary material for: Long Noncoding RNA LINC00941 Links Oncogenic KRAS Signaling to Aggressiveness, DNA Repair, and Chemoresistance in Pancreatic Cancer
Source: IUBMB Life. 2026 Nov 9;78(11):e70140. doi: 10.1002/iub.70140 (PMC13748920; doi:10.1002/iub.70140)
Supplement: Supplementary file 6 [file IUB-78-0-s009.pdf]

Supplementary Table 1. Primers used for qRT-PCR.

Gene	RefSeq transcript ID targeted	Forward primer (5′–3′)	Reverse primer (5′–3′)	Amplicon size (bp)	Use
LINC00941	NR_040245.1	TTTTGTGTCCAAGCCCCAGA	TGGGAGACTGAGATGGAAGG	150	Target
AC006262.5	NR_135234.1	TCCTCAGCCCAGCCGAAG	AGAGGGTGTTCACATTGCTTT	105	Target
KRAS	NM_004985.5	CCCAGGTGCGGGAGAGA	CAGCTCCAACTACCACAAGTTT	63	Target
BACH1	NM_001186.4	GAACCCCAGCAAGAACCTTG	TCCGTTGTGCATTGAATGGC	142	Target
TERF2IP	NM_018975.4	CCTCCTCCCAGAAGCTCAAG	CAGGCAAATCTGGAGTTCTCT	93	Target
CDK7	NM_001799.4	GCACACCAACTGAGGAACA	TCTCCTGCTGCACTGAAGAT	109	Target
ATRX	NM_000489.6	AGCATTAAGTAGACAAGCCAGC	CTGATTGTACTGCTGCTGGA	145	Target
TOP2A	NM_001067.4	TGGTGGCAAGGATTCTGCTA	CATTCAGGCTCAACACGCTG	135	Target
BIRC5	NM_001168.3	GGACCACCGCATCTCTACAT	GAAACACTGGGCCAAGTCTG	130	Target
SPIDR	NM_001282919.1	AGAATGCCCATCCTTTCCAG	AGGTGCTTTTCTGTAATCGTC	140	Target
PPP4C	NM_002720.3	ATGACCTCAAAGAGCTGTTCAG	TAGAAGCCACGGTCCACAAAG	88	Target
ABCG2	NM_001348986.2	ACTTTGGGCTAAAAAATGATTCTACTG	CACTGGTTGGTGGTCAGGAA	76	Target
GAPDH	NM_002046.7	GAGCCGCATCTTCTTTTGC	CCATGGTGTCTGAGCGATGT	54	Reference gene
HMBS	NM_000190.4	GGCAATGCGGCTGCAA	GGGTACCCACGCGAATCAC	64	Reference gene
