# Supplementary material for: Long Noncoding RNA LINC00941 Links Oncogenic KRAS Signaling to Aggressiveness, DNA Repair, and Chemoresistance in Pancreatic Cancer
Source: IUBMB Life. 2026 Nov 9;78(11):e70140. doi: 10.1002/iub.70140 (PMC13748920; doi:10.1002/iub.70140)
Supplement: Supplementary file 7 [file IUB-78-0-s010.pdf]

SUPPLEMENTARY FIGURE 3

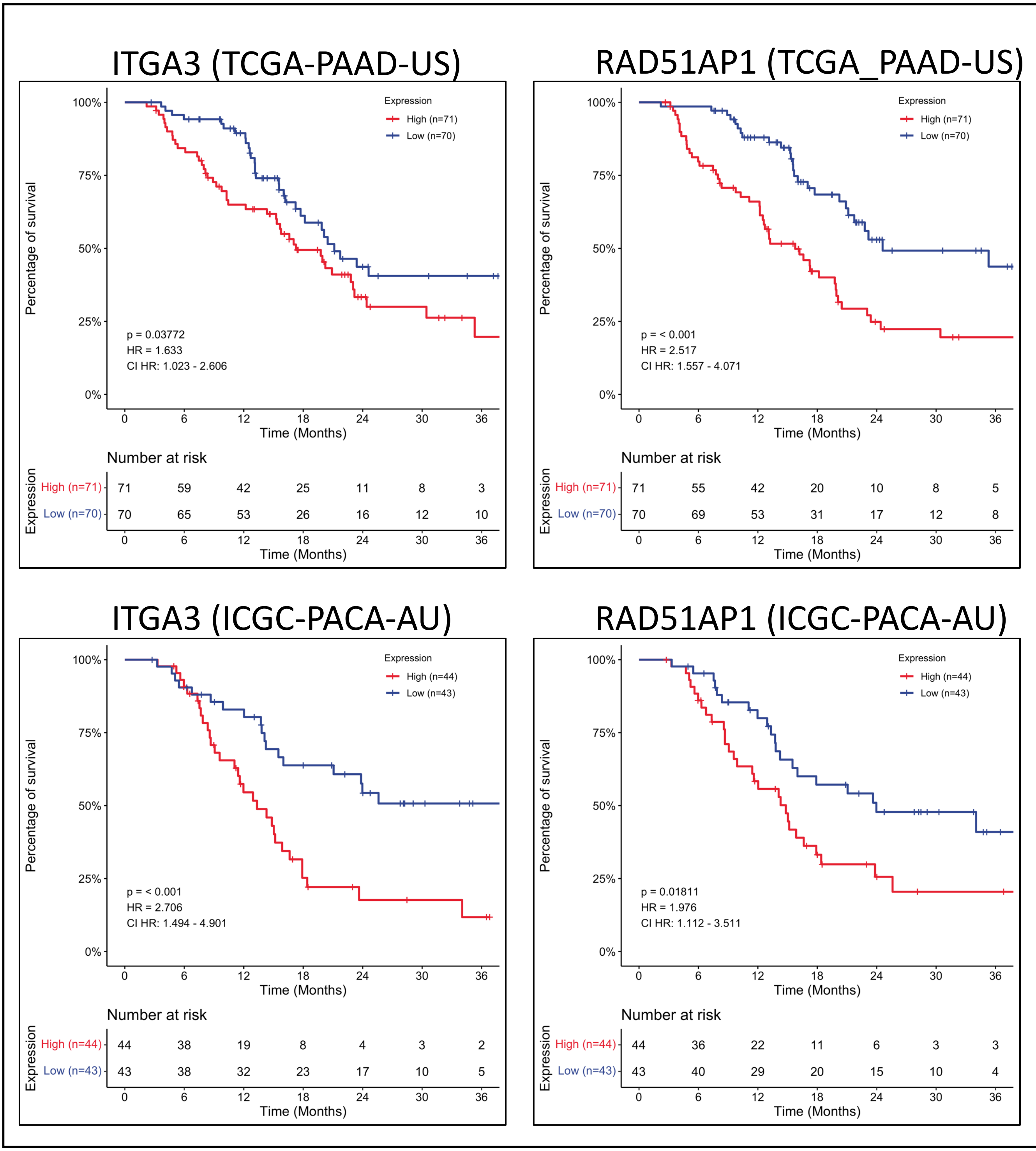

High expression of LINC00941-correlated genes in the TCGA cohort ITGA3 and RAD51AP1 is associated with poor survival in PDAC. Kaplan–Meier survival analysis based on ITGA3 (left) and RAD51AP1 (right) expression in the TCGA (PAAD-US, n = 145) and ICGC (PACA-AU, n = 87) cohorts as indicated. Patients were stratified into high- and low-expression groups using the median expression cutoff. Differences in survival were evaluated using the log-rank test; log-rank *p*-values and hazard ratios (HR) with 95% confidence intervals are shown.
